# Constellation of the endophytic mycobiome in spring and winter wheat cultivars grown under various conditions

**DOI:** 10.1038/s41598-023-33195-y

**Published:** 2023-04-13

**Authors:** Sylwia Salamon, Katarzyna Mikołajczak, Lidia Błaszczyk

**Affiliations:** Department of Plant Microbiomics, Institute of Plant Genetics PAS, Poznan, Poland

**Keywords:** Ecology, Microbiology, Plant sciences

## Abstract

The mycobiome is an integral component of every living organism. Among other fungi associated with plants, endophytes are an interesting and favorable group of microorganisms, but information regarding them is still largely unknown. Wheat is the most economically significant and essential crop for global food security, which is exposed to a range of abiotic and biotic stresses. Profiling plants’ mycobiomes can help in sustainable, chemical-reducing wheat production. The main objective of this work is to understand the structure of endogenous fungal communities in winter and spring wheat cultivars growing under different growth conditions. Further, the study attempted to investigate the effect of host genotype, host organs and plant growth conditions on the composition and distribution of fungi in wheat plant tissues. Comprehensive, high throughput analyzes of the diversity and community structure of the wheat mycobiome were performed, complemented by the simultaneous isolation of endophytic fungi, resulting in candidate strains for future research. The findings of the study revealed that the type of plant organs and growth conditions influence the wheat mycobiome. It was also assessed that fungi representing the genera *Cladosporium*, *Penicillium*, and *Sarocladium* form the core mycobiome of Polish spring and winter wheat cultivars. The coexistence of both symbiotic and pathogenic species in the internal tissues of wheat was also observed. Those commonly considered beneficial for plants can be used in further research as a valuable source of potential biological control factors and/or biostimulators of wheat plant growth.

## Introduction

The plant holobiont consists of microbial consortia (fungi, bacteria, archaea, and protists) that live within (endosphere), on the aboveground (phyllosphere), and underground (rhizosphere) organs of plant. Endogenous microbial communities (endophytes) contribute to mitigating abiotic^1^ and biotic stresses^2^ and promote host plant growth^3^. Further study of the plant microbiome is crucial due to its impact on host health and fitness. These research can also be utilized to design the microbiome to enhance plant tolerance to unfavorable environmental conditions^4^.

Wheat (*Triticum aestivum* L.) is the most economically significant and essential crop for global food security. Increasing wheat tolerance to biotic and abiotic stresses and intensifying crop yields without the use of chemicals can help achieve sustainable crop production. Since microorganisms play a significant role in plant health and growth, the introduction of naturally occurring, beneficial microorganisms into wheat breeding could provide a solution. Paradoxically, the wheat plants themselves are an excellent source of eligible microorganisms. The endosphere of wheat is rich in fungi that have several functions. Some of these functions, including their antagonistic action against phytopathogens^5^, improvement of plant tolerance to abiotic^6^ and biotic stresses, or promotion of wheat growth and yield^7,8^ have been recently summarized by us^9^.

For a decade, intensive research has been conducted on the wheat mycobiome, due to the undeniable importance of endogenous fungi on this cereal. In addition, the increased availability of a high-throughput culture-independent molecular approach (ITS metabarcoding) has supported a deeper study of wheat. The culture-independent or culture-dependent methods were used to assess whether the wheat microbiomes were affected by the following: host genotype^10–14^, location^10,12,14,15^, management strategies^15–18^, growth stages^12,16,17,19^, leaf position^10^, host organ^11,12,14,17^, nitrogen input in the soil^19^, Fusarium head blight disease^20^, and water stress history^13^. However, a comprehensive evaluation of the diversity and overall composition of endogenous fungi across wheat cultivars and forms, individual wheat organs, cultivation strategies using both conventional culture-dependent and high-yield techniques has not yet been documented. In general, compared to bacterial endophytes, information on the wheat mycobiome, especially on fungi associated with wheat internal tissues, is still scarce. Therefore, it is necessary to intensify work on the identification of beneficial wheat endophytic fungi that could be used as biocontrol agents or plant growth promoters.

In order to meet these needs, the main objective of the presented research was to analyze the structure of endogenous fungal communities of ten cultivars of spring and winter wheat in various conditions of their growth, i.e.: conventional field with mineral fertilization and herbicides, but without fungicides; no-till field without mineral fertilization, herbicides and fungicides; under controlled conditions in a greenhouse with mineral fertilizer. The culture independent ITS metabarcoding was used to investigate the effects of the host genotype, its organs, and growth conditions on the composition and distribution of fungi in wheat plants. Additionally, organ-, genotype- and growth conditions-specific fungi were isolated and selected using a culture-dependent approach to provide new fungal strains for future exploration of growth-promoting and biological control agents. Endogenous core wheat mycobiome was also determined.

## Results

A total of 220 fungal operational taxonomic units (OTUs) and 726 endogenous fungal isolates were obtained. The OTUs that had at least five sequences in all the samples were used in the analysis. The data obtained by the metabarcoding approach were deposited in Sequence Read Archive (SRA) under the Bio Project PRJNA899455. The DNA sequences from the culture-dependent method were deposited in the NCBI GenBank database (Table S1). The combined results from both the approaches revealed that the *Ascomycota* type was the most common in the wheat endosphere in all analyzed groups. Fungi representing the second most abundant *Basidiomycota* type were found mostly in the roots. *Glomeromycota,* considered to be a phylum of beneficial arbuscular mycorrhizal fungi, have been mainly observed in conventional field cultivation systems in two wheat cultivars.

### Culture-independent ITS2 metabarcoding

#### Alpha diversity

**Figure 1:**
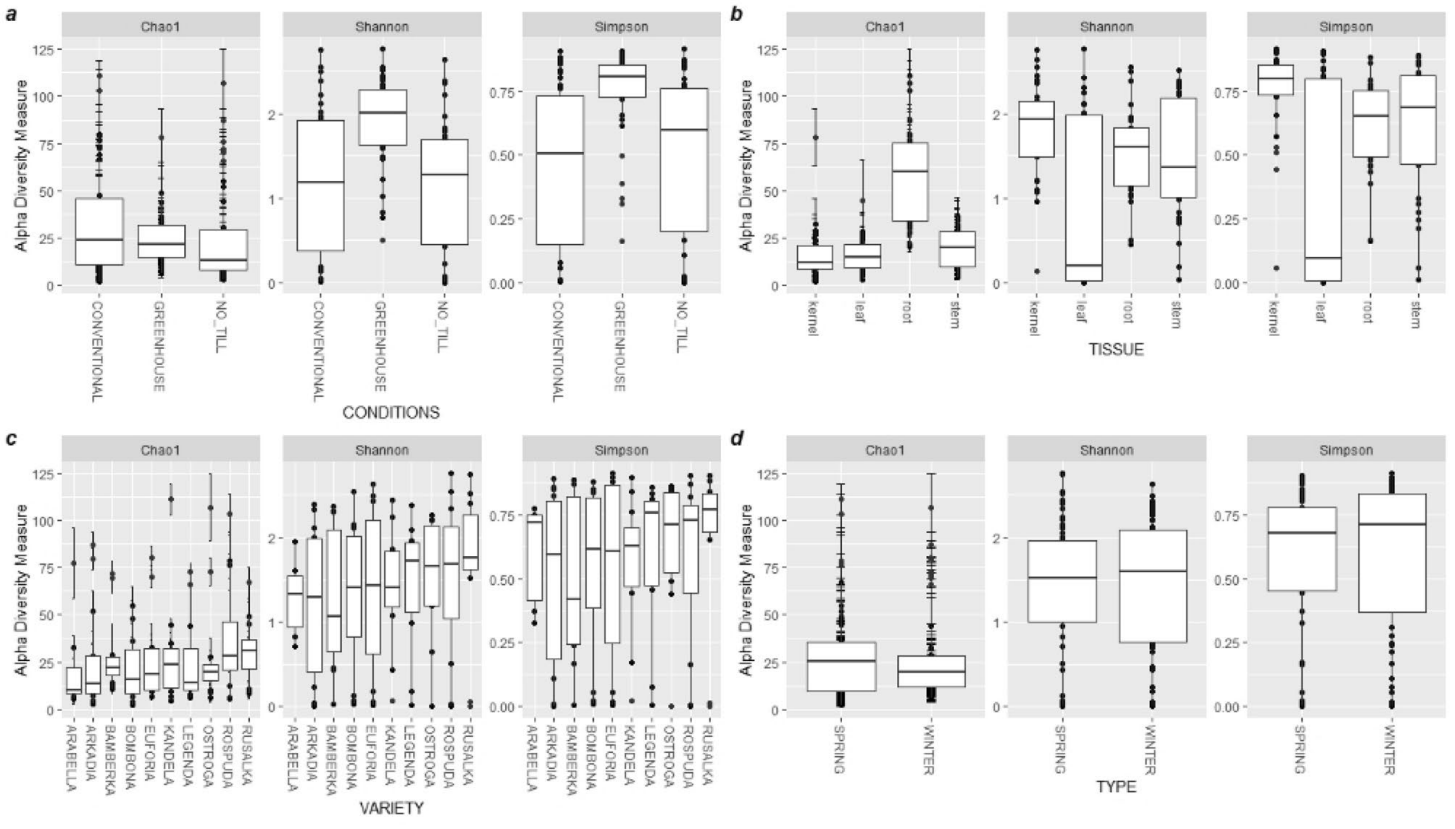
Alpha diversity estimates of endogenous fungi in wheat plants containing the Chao, Shannon and Simpson indices evaluation and separated based on (**a**) growth conditions, (**b**) tissue types, (**c**) cultivars, and (**d**) wheat forms by performing the Wilcoxon rank sum test using pairwise comparisons.

The endogenous fungal diversity was significantly higher in plants from the greenhouse compared to those from the field conditions (*p* < 0.0, Fig. 1). The endogenous fungal diversity was generally lower in leaves than in the kernels (*p* < 0.01), the stems (*p* < 0.05), and roots (*p* < 0.05). Further, the species richness was significantly higher in the roots compared to other wheat tissues (*p* < 0.01). Though the fungal alpha diversity was generally consistent across the varieties and forms, Rusałka and Bamberka demonstrated the highest and the lowest diversity, respectively, in the studied cultivars (Fig. 1).

#### Beta diversity

**Figure 2:**
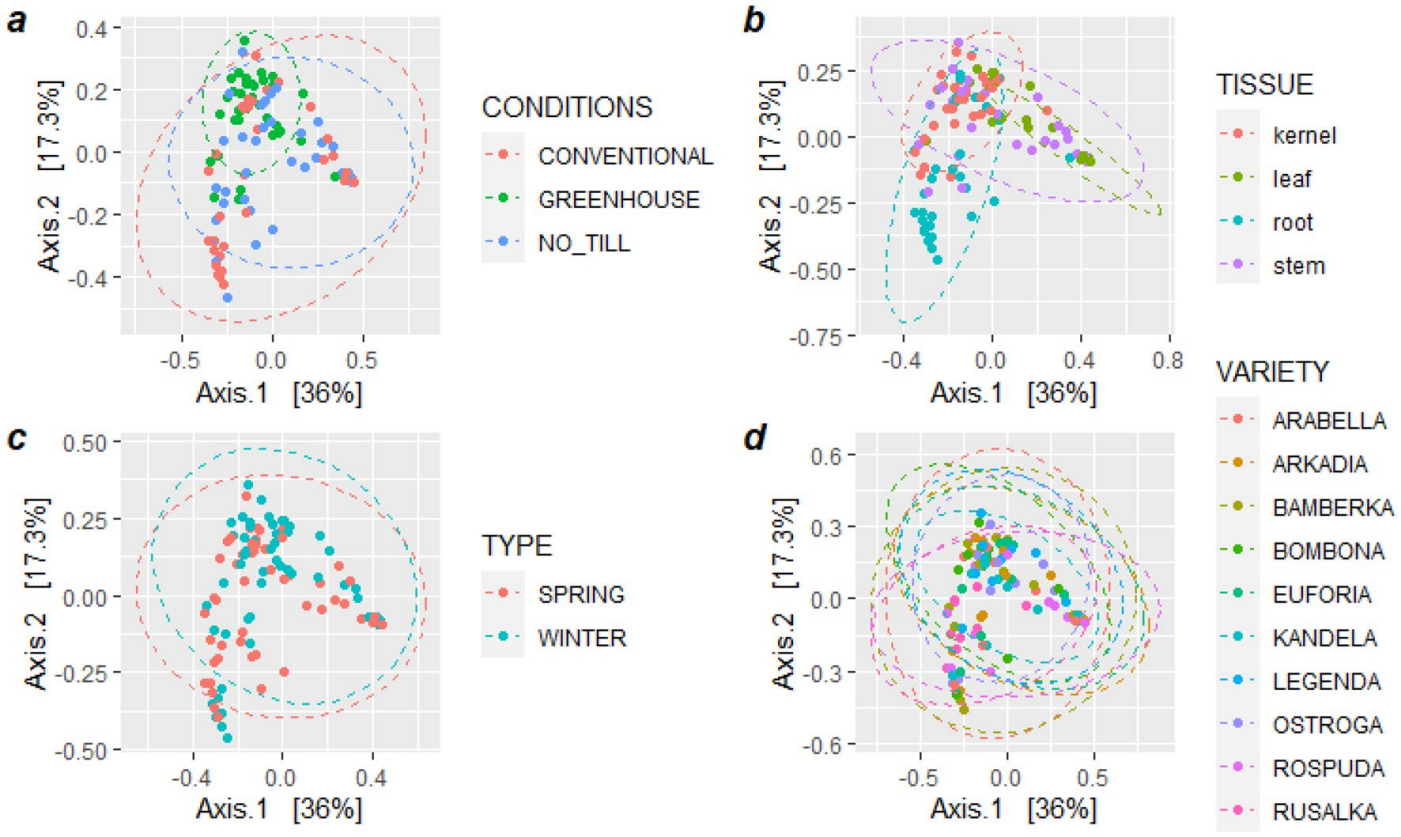
PCoA plots based on weighted UniFrac of the fungal community structure of wheat endosphere, depending on (**a**) growth conditions, (**b**) tissue types, (**c**) wheat forms, and (**d**) cultivars.

Based on the weighted UniFrac distance, principal coordinates analysis (PCoA) was used to compare biological communities across plant growth management types, organs, cultivars, and forms (Fig. 2). OTUs from the greenhouse were slightly separated, whereas OTUs from conventional and no_till management conditions were clustered together (Fig. 2a).

**Figure 3:**
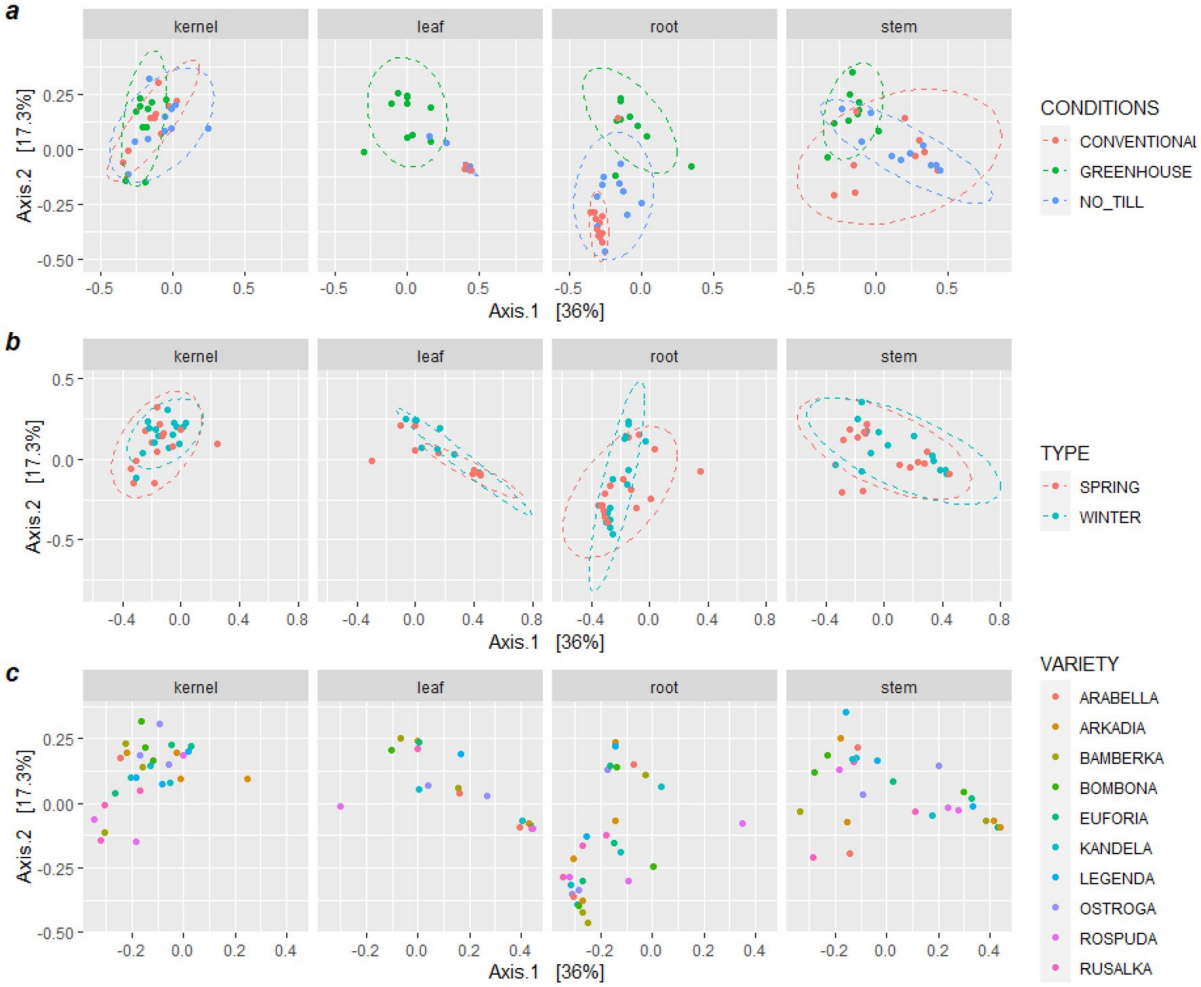
PCoA based on weighted UniFrac of the endogenous fungal communities’ structure in diverse wheat (**a**) cultivars, (**b**) conditions, and (**c**) forms depending on wheat tissue.

Moreover, OTUs from the roots were clearly separated from each other, while stems, kernels, and leaves were clustered together (Fig. 2b). Applied growth conditions mainly affected the mycobiome of the wheat roots, leaves and stems (Fig. 3a). Samples from 2 wheat forms and 10 cultivars clustered together (Fig. 2c,d). The PERMANOVA analysis on weighted UniFrac distance revealed that the growth management strategy type and part of the plant tissue significantly impacted the endogenous fungal community in wheat (*p* < 0.001).

**Figure 4:**
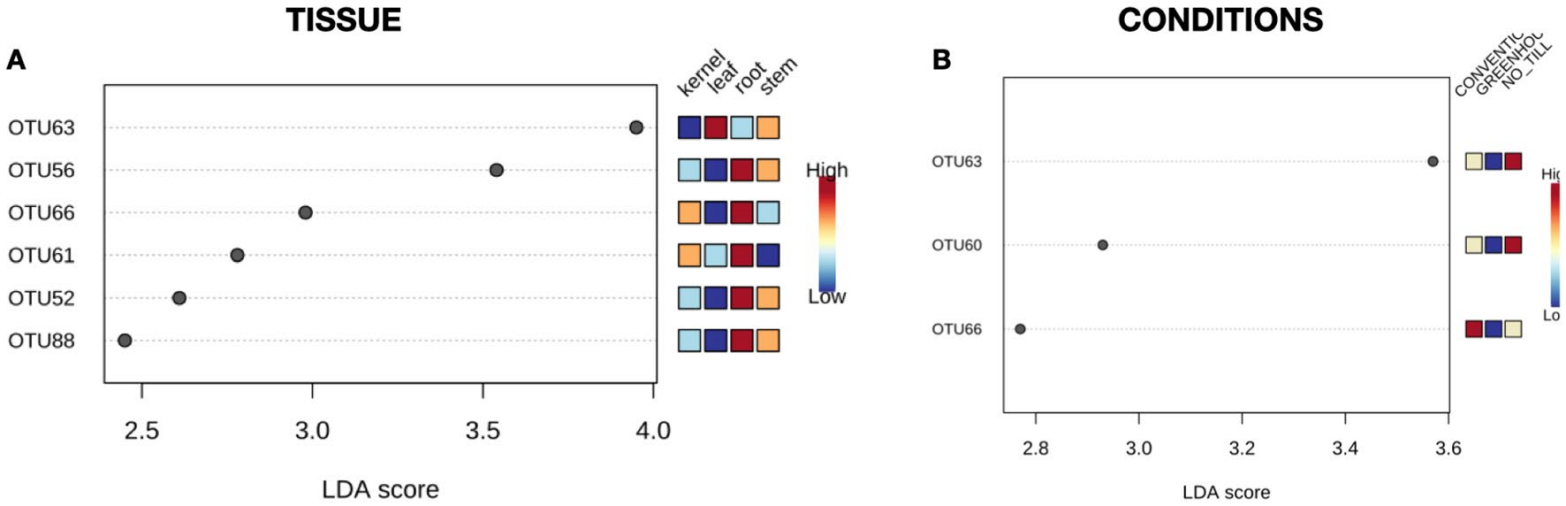
OTU with significant differential abundance regarding (**A**) wheat tissues and (**B**) growth conditions, created by Linear Discriminant Analysis Effect Size (LEfSe). The heat maps indicate the OTU abundance in each group.

The *adonis* tests and the PCoA observation found that the tissue type explained approximately 25% (R2 (wUniFrac) = 0.25063) of the variation of communities in the dataset, while growth conditions account for 9% (R2 (wUniFrac) = 0.0926) of the variation. Betadisper demonstrated that „conditions” and „tissue” groups had a similar dispersion (Conditions: F = 1.9771, p = 0.13; Tissue: F = 2.3828, p = 0.073), thus the observed significant differences were not a result of heterogeneity of dispersions. OTUs with significantly different abundances in various tissue types and growth conditions, were identified and demonstrated in Fig. 4. The wheat cultivars (p = 0.979) and forms (p = 0.389) had an insignificant effect on the fungal community structures in the entire data set (Fig. 2c,d), and on individual tissues (Fig. 3b,c).

#### The abundance of fungal endophytes in wheat

The level of fungal colonization in the inner part of the wheat plants was lowest in greenhouse samples (mean per sample =  ~ 1359 ITS copies) compared to the conventional and no_till plant groups (mean per sample =  ~ 23,530 and ~ 29,660 ITS copies, respectively). The most abundant in endogenous fungi were the roots, while the kernels and stems demonstrated a low level of endophytes colonization. The winter wheat forms as well as Arkadia and Bamberka manifested the highest endogenous fungal abundance.

#### Endogenous fungal community composition

**Figure 5:**
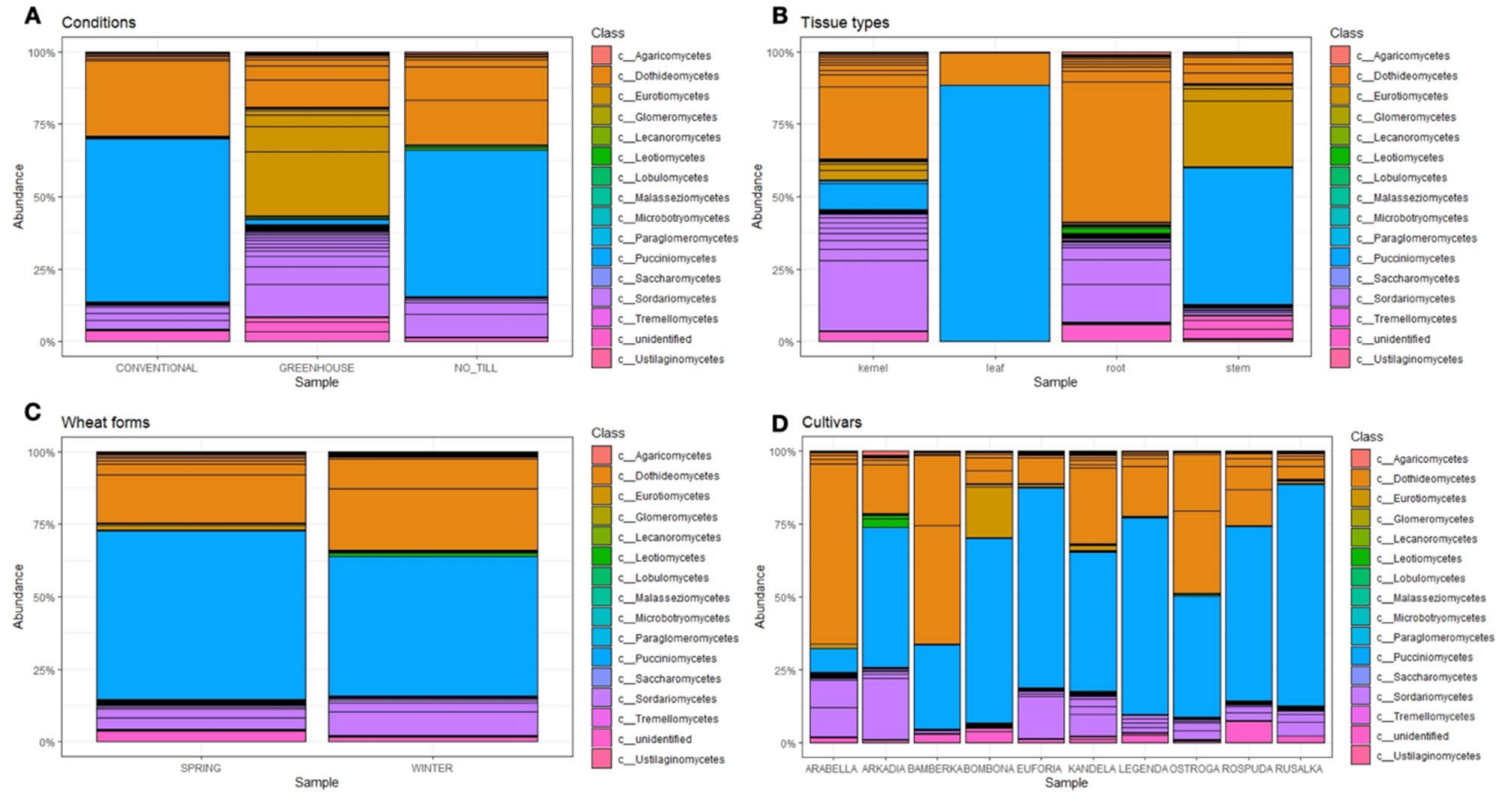
Relative abundance of endogenous fungi of wheat from diverse (**A**) conditions, (**B**) tissue types, (**C**) forms, and (**D**) cultivars on a class level.

**Figure 6:**
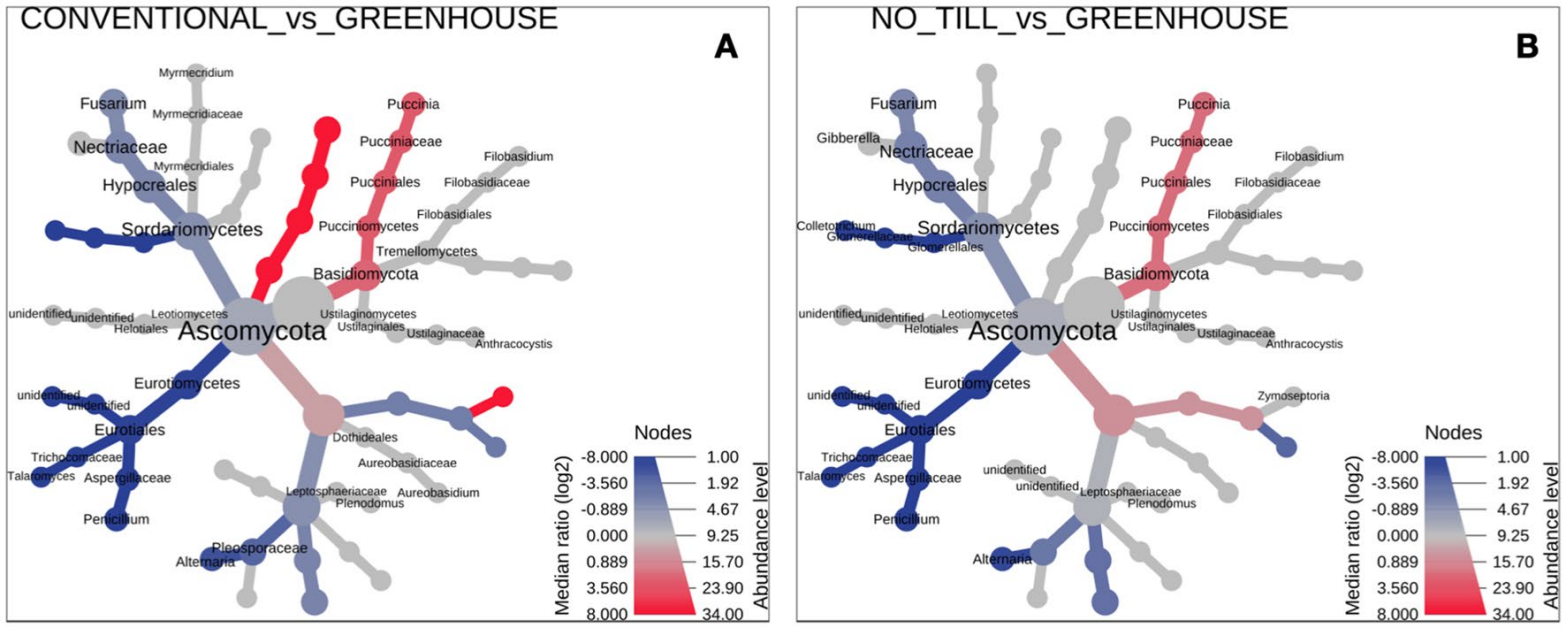
Heat tree visualization of taxonomic differences of endogenous fungi in various growth conditions. Blue and red nodes indicate the lower and higher abundance, respectively, compared to control. (**A**) Taxonomic differences in wheat cultivated in conventional conditions compared to the control plants (greenhouse). (**B**) Taxonomic differences in wheat cultivated in no_till conditions compared to the control plants (greenhouse).

**Figure 7:**
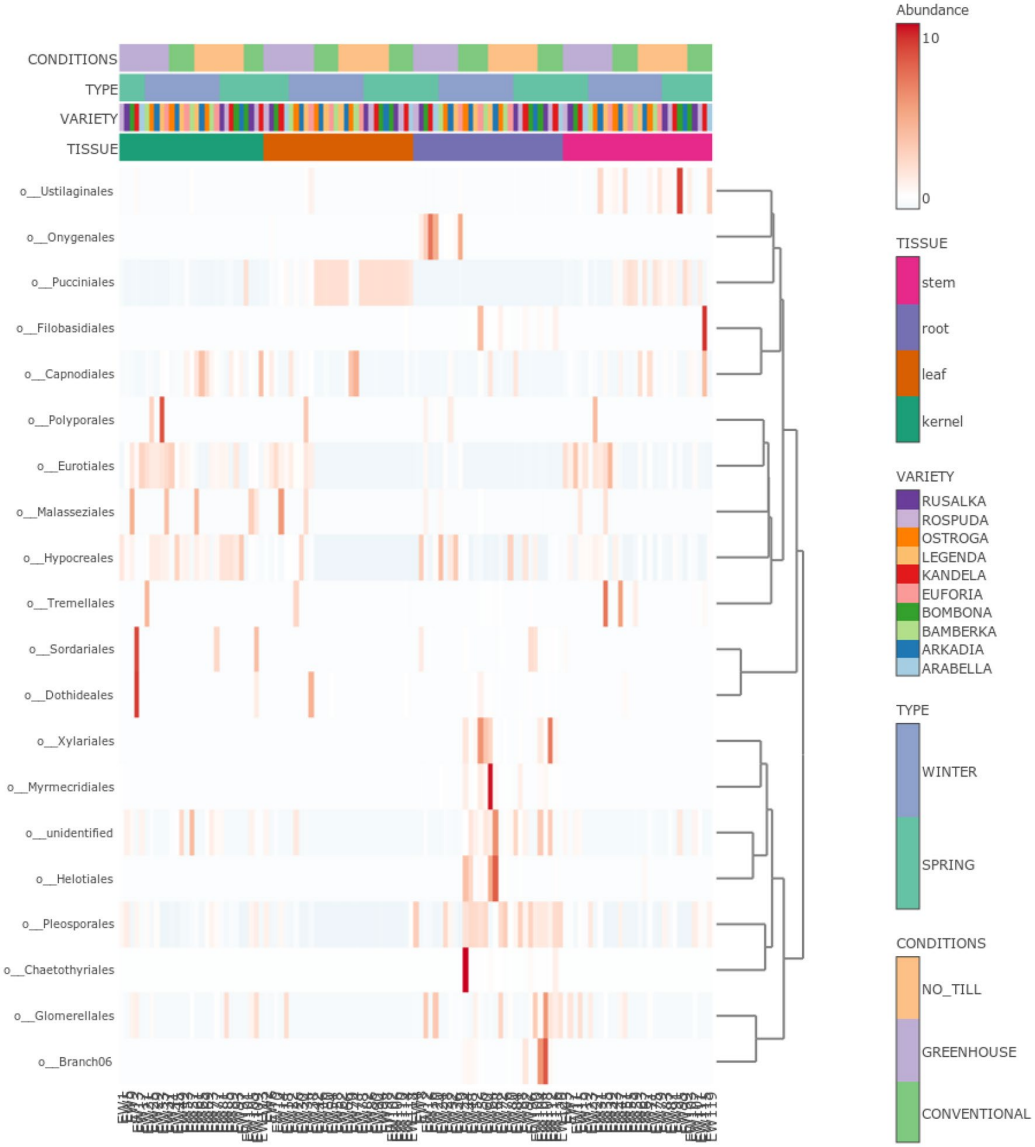
Heat map presenting the abundance of endogenous fungi of wheat in diverse conditions, tissue types, forms, and cultivars on order level. The color gradient (white to red) represents the fungal abundance level in each sample.

The fungal member classes of *Dothideomycetes* and *Sordariomycetes* dominated the *Ascomycota* phylum in all the analyzed groups (Fig. 5). Furthermore, the *Pucciniomycetes* class (*Basidiomycota* phylum) has become widespread in the leaves from field plants. Interestingly, these fungi were also identified in the stems and kernels of the studied plants. The composition of the endogenous fungal communities differs among the analyzed growth conditions (Figs. 5, 6, 7). In the conventional and the no_till samples, the *Pleosporales* and *Pucciniales* orders were the most dominant. In the greenhouse samples, there was an advantage of the *Eurotiales*, with *Hypocreales*. The *Pucciniales* fungi were observed exclusively in field-collected plants (conventional and no_till). Further, the *Chaetothyriales*, *Dothideales*, and *Xylariales* fungi were absent in the greenhouse samples, though present in the field-collected samples. However, *Conioscyphales*, and *Thelebolales* were identified mostly in the greenhouse samples, whereas *Onygenales* order and *Malasseziomycetes* classes were absent in the no_till field samples. *Glomeromycota* phylum was identified mainly in the conventional samples (*Glomeromycetes* and *Paraglomeromycetes* classes) and in smaller amounts in no_till field conditions (*Paraglomeromycetes).*

The clear differences in endophytic fungal community structure were also observed between studied wheat tissue (Figs. 5, 7).

The *Capnodiales* order, mainly the *Cladosporium* genus, dominated strongly in the leaf tissue. The *Chaetothyriales*, *Conioscyphales*, *Dothideales*, *Helotiales*, *Myrmecridiales*, *Onygenales*, *Thelebolales* and *Xylariales* orders predominantly or exclusively existed in the wheat roots, while the *Sordaliales* order was present in the roots, kernels, and stems. Furthermore, the fungi belonging to the *Pleosporales* order colonize a large amount of wheat endosphere in all the analyzed cultivars (Fig. 7). Additionally, the *Eurotiales* occurrence was the highest in the Bombona cultivar. Bamberka and Ostroga demonstrated a large level of the *Cantharellales* fungi. The *Erysiphales* and the *Magnaporthales* orders were exclusively observed in the endosphere of the winter wheat forms. However, *Conioscyphales* were observed mainly in the spring wheat plants.

### Culture-dependent barcoding

As a result of the isolation of the endogenous fungi from 119 samples, 726 isolates were obtained and molecularly identified. The strains were identified at the genus or the species level. All identified taxa were listed in Table 1.

**Table 1:** Endogenous fungal taxa isolated from roots, leaves, stems and kernels of wheat cultivated in conventional field, no_till field and controlled greenhouse conditions.

Fungal taxa	Growth conditions			Tissue type			
	Greenhouse	Conventional	No_till	Roots	Leaves	Stems	Kernels
*Achroiostachys* sp.	–	X	–	–	–	X	X
*Acremonium sclerotigenum*	X	–	–	X	–	–	X
*Acremonium* sp.	X	–	–	–	X	–	–
*Akanthomyces* sp.	–	X	–	X	–	–	–
*Alternaria alternata*	–	X	X	–	–	X	X
*Alternaria conjuncta*	–	X	–	–	X	–	–
*Alternaria hordeicola*	–	–	X	–	–	X	–
*Alternaria infectoria*	–	X	X	–	X	–	X
*Alternaria rosae*	–	X	–	–	X	–	–
*Alternaria* sp.	–	X	X	X	X	X	X
*Alternaria tenuissima*	–	X	–	–	X	–	–
*Anthracocystis flocculosa*	–	X	X	–	X	–	X
*Anthracocystis* sp.	–	X	X	–	X	–	X
*Arthrinium* sp.	–	X	–	X	–	–	–
*Aspergillus* sp.	X	–	–	–	X	–	X
*Aureobasidium pullulans*	–	X	X	–	X	X	–
*Backusella* sp.	–	X	–	X	–	–	–
*Bipolaris sorokiniana*	–	X	–	X	–	–	–
*Cadophora* sp.	–	X	–	–	–	X	–
*Chaetomium* sp.	–	X	–	X	–	–	–
*Chrysosporium pseudomerdarium*	X	–	–	–	–	X	–
*Cladosporium allicinum*	–	X	–	–	X	–	–
*Cladosporium cladosporioides*	X	–	–	–	X	–	–
***Cladosporium* sp.**	**X**	**X**	**X**	**X**	**X**	**X**	**X**
*Clonostachys candelabrum*	X	–	–	–	–	–	X
*Didymella pomorum*	–	X	–	–	–	–	X
*Didymella* sp.	–	–	X	–	–	–	X
*Engyodontium album*	X	–	–	–	–	–	X
*Epicoccum* sp.	–	X	X	X	–	–	–
*Fusarium avenaceum*	–	X	X	X	–	–	–
*Fusarium oxysporum*	–	X	X	X	X	X	X
*Fusarium poae*	–	X	–	X	–	–	–
*Fusarium proliferatum*	X	–	–	X	X	–	–
*Fusarium redolens*	–	X	–	X	–	–	–
*Fusarium solani*	–	X	X	X	–	X	–
***Fusarium* sp.**	**X**	**X**	**X**	**X**	**X**	**X**	**X**
*Fusarium temperatum*	–	–	X	–	–	–	X
*Geomyces pannorum*	X	–	–	–	–	X	–
*Gibellulopsis* sp.	–	–	X	–	–	–	X
*Gloeotinia* sp.	–	–	X	–	–	X	–
*Isaria farinose*	–	X	–	X	–	–	–
*Leptobacillium leptobactrum*	–	–	X	–	–	X	–
*Marasmius* sp.	X	–	–	–	–	–	X
*Meyerozyma* sp.	–	–	X	X	–	–	–
*Microdochium bolleyi*	–	X	X	X	–	X	–
*Microdochium* sp.	–	X	–	X	–	–	–
*Moesziomyces bullatus*	–	–	X	–	–	X	–
*Moesziomyces* sp.	-	X	X	–	X	–	X
*Neonectria* sp.	–	X	–	X	–	–	–
*Nigrospora gorlenkoana*	X	X	–	X	X	–	–
*Penicilium amphipolaria*	–	X	–	–	X	–	–
***Penicillium chrysogenum***	**X**	**X**	**X**	**X**	**X**	**X**	**X**
*Penicillium crustosum*	X	X	–	–	X	–	X
*Penicillium digitatum*	X	–	–	X	X	–	X
*Penicillium expansum*	X	–	–	–	–	–	X
*Penicillium olsonii*	X	–	X	X	X	X	X
***Penicillium* sp.**	**X**	**X**	**X**	**X**	**X**	**X**	**X**
*Periconia macrospinosa*	–	X	X	X	–	X	–
*Periconia* sp.	–	X	–	X	–	–	–
*Phlebia* sp.	X	–	–	–	–	X	–
*Phoma eupyrena*	–	X	–	X	–	–	–
*Phoma pomorum*	–	X	X	X	–	–	–
*Phoma* sp.	–	X	–	X	X	–	–
*Plectosphaerella cucumerina*	–	–	X	X	–	-	–
*Rhizoctonia solani*	–	X	–	–	–	X	–
***Sarocladium* sp.**	**X**	**X**	**X**	**X**	**X**	**X**	**X**
*Sarocladium strictum*	X	X	–	X	X	X	X
*Setophoma terrestris*	–	X	X	X	–	X	–
*Setosphaeria pedicellata*	–	X	X	X	–	X	–
*Stemphylium vesicarium*	–	X	–	–	–	X	–
*Talaromyces aculeatus*	–	X	–	–	–	X	–
*Trichoderma hamatum*	X	–	–	X	–	–	–
*Trichoderma koningii*	X	–	–	–	–	–	X
*Trichoderma* sp.	X	–	X	X	–	–	X
*Trichoderma viride*	X	–	-	X	––	–	X
*Umbelopsis* sp.	X	–	–	X	–	–	–
*Waitea circinata*	–	X	X	X	–	–	–

**Figure 8:**
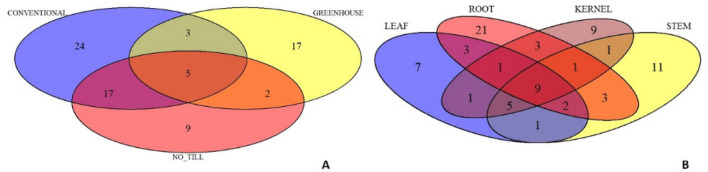
Relationship of fungal community composition between the studied (**a**) management strategies and (**b**) wheat organ.

Figure 8 demonstrates the comparison between the studied management strategies and the analyzed plant organs of the isolated fungi. Endogenous fungi of wheat isolated from all the growth conditions studied (greenhouse, conventional, and no_till in the field) are fungi that belong to *Cladosporium*, *Penicillium*, *Sarocladium*, *Fusarium* genera, and *Penicillium chrysogenum* species. The species isolated from the greenhouse plants differ from those grown in field conditions. Only, *Nigrospora gorlenkoana*, *Penicillium crustosum*, and *Sarocladium strictum* were isolated from both the greenhouse and conventional conditions, while *Penicillium olsonii* and *Trichoderma* sp. were the common fungi found in plants grown both in the greenhouse and no_till field conditions. Meanwhile, 17 taxa of fungi were distributed within conventional and no_till conditions (Fig. 8a, Table 1). Unique species were also observed in each of these groups, with lower fungal diversity in plants from the no-till conditions (9 unique taxons, Table 1) than in the conventional managed plants (24 unique taxons:, Table 1).

Additionally, tissue-specific endogenous fungi were also observed (Fig. 8b). The highest number of unique taxa of endogenous fungi was noted in the roots (21 taxons), 9 kernels-specific and 7 leaves-specific fungi were detected (Table 1). Only 9 endophytes were common in all the studied organs: *Alternaria* sp., *Fusarium* sp., *Fusarium oxysporum*, *Penicillium* sp., *Penicillium chrysogenum*, *Penicillium olsonii*, *Sarocladium* sp., *Sarocladium strictum*, and *Cladosporium* sp.

## Discussion

Five winter and five spring wheat cultivars were grown in conventional and no_till management on the field, and in controlled conditions in the greenhouse. The study used culture-independent and culture-dependent methods to identify a large amount of co-occurring endogenous fungi, representing different lifestyles and colonizing internal parts of wheat plants. These observations are consistent with previous studies^11,18^. Present research identified fungi of the genera *Alternaria, Acremonium, Cladosporium, Phoma, Chaetomium, Fusarium,* and *Epicoccum*, which have been described as wheat endophytes in the literature^14,18,21–24^. There were beneficial fungi, which can potentially be used in biological plant protection, observed among the identified endophytes. The species *Aureobasidium pullulans* were identified in almost all the studied wheat cultivars (except cv. Arabella). The growth suppression of post harvested plants pathogens was affected by the high enzymatic activity of *A. pullulans*^25^. The enzymes produced by this fungus suppress the growth of phytopathogens, thereby helping in the biological control of plant diseases. It was found that this species is the source of antimicrobial compounds, such as 8,9-dihydroxy-2methyl-4H,5H-pyranol[3,2-c] chromen4-one, 2-propyloacrylic acid, hexane-1,2,3,4,5,6-hexol, and 2-methylenesuccinic acid^26^. *Auerobasidium pullulans* has been found to be effective against *Botrytis cinerea*^27,28^, *P. expansum*^29,30^, and *Rhizopus stolonifer*^27^. The present study isolated a few representatives of *Trichoderma* genus, such as *T. koningii,* which can reduce the severity of the disease in wheat caused by *P. triticis repentis* and *Mycosphaerella graminicola*^31^, as well as *B. sorokiniana,* and *A. alternata*^32^. *Trichoderma hamatum*, identified in the roots of the studied plants, demonstrated the antagonistic effect against *Pyrenophora tritici-repentis*—the causal agent of the tan spot in wheat^5^. Meanwhile, *T. atroviride* identified in this study is widely known to be beneficial to plants and as a mycopathogen that can be used to block wheat diseases caused by pathogenic fungi^33^. The work also isolated the *Chrysosporium pseudomerdarium*, which is known to produce gibberellins and support the host plant growth^34^. It can therefore be concluded that the results presented in this paper have highlighted the importance of wheat plants as a source of beneficial microorganisms. However, further studies are needed to prove the biological role of the strains isolated here in relation to wheat plants and its pathogens, to consider their use in biological control or as promoters of plant growth or resistance. This study also captured fungi, commonly referred to as wheat pathogens, such as *Fusarium* sp. (*F. poae*, *F. oxysporum*); *Arthrinium* sp. and *Colletotrichum* sp. or *B. sorokiniana*, *Microdochium bolleyi*, and *R. solani.* The relationship of these fungi with wheat was described in previous reports^35–39^. Therefore, the coexistence of pathogens and beneficial microbes in the plant organism is possible, although the key to their health is microbiome balance and equality^40^. This is an extremely unstable state because, as Manzotti^41^ have shown isolation and reinfection of the same host plant with strains of pathogenic species can cause disease symptoms in the plant and introduce a state of dysbiosis. Nevertheless, the roles of most of the observed fungi inhabiting the endosphere of the studied wheat plants, such as *Penicillium digitatum*, *Penicillium crustosum*, *Engyodontium album*, *Cladosporium cladosporioides, Nigrospora gorlenkoana, Setophoma terrestris, Setophaeria pedicellata, Periconia macrospinosa, Stemphylium vesicarium, Anthracocystis flocculosa*, are unknown, and further study on these fungi is required. *Penicillium digitatum* is commonly found in the soils of citrus plants and infects the fruits through biotic or abiotic factors in the fields, or during harvesting and postharvest processing^42^. Another species of the *Penicillium* genus, namely *P. crustosum*, is commonly found on cheeses and nuts. However, this species is also a postharvest pathogen that causes pome and stone fruit blue mold disease in the plants^43,44^. This species produces mycotoxins, such as penitrem A, roquefortine C, terrestrial acid, and cyclophenol, which are hazardous to human health^45^. *Engyodontium album* is a common species that are found in several environments, including extreme environments, such as polar soils and environments with high salinity. Further, this fungus was often isolated from various cultural heritage materials, such as stone, wood, glass, or paper^46^. It has also been documented that *P. album* can feed on dead arthropods, especially spiders. *Cladosporium cladosporioides* is also a common and cosmopolitan saprophyte^47^. This species has been detected as a secondary invader on the necrotic parts of a variety of host plants. According to reports, *C. cladosporioides* is involved in several human health problems, including pulmonary and cutaneous infections. Though *Nigrospora gorlenkoana* is not studied widely, previous reports recognize it as a plant pathogen isolated from the fruits and leaves of *Vitis vinifera*^48^. Meanwhile, *S. terrestris* has been found to cause pink root rot in pumpkin, oilseed rape, and onion^49,50^. *Periconia macrospinosa* has previously been reported as a beneficial endophyte. However, the species has been found to be a pathogen causing necrosis in some cases, such as the leaf necrosis of the ogival gourd found in India^51^. The next endophytic fungus isolated from the studied wheat plants is *Stemphylium vesicarium*. This fungus is also known as a pathogen that causes important foliar diseases of *Allium* spp., and a range of vegetable, field, ornamental, and woody crops^52^. *Anthracocystis flocculosa* is a species of yeast, which has been described as a foliar epiphyte of *Trifolium pratense* L. The species is commonly known as *Pseudozyma flocculosa*^53^, which produces the antifungal glycolipid, flocculosin, unique to this fungus. It is also responsible for antibiotic activity against powdery mildews^54,55^. This wealth of genera, species and abundance of strains obtained from the endosphere of ten varieties of wheat, with the present knowledge of the mode of life of these fungi, which in addition can change, falls under the last terminology "endophytes" or "endophytic fungi", as those that inhabit plant endosphere^14^. Since the role of most of these fungi in wheat plants is unknown, work is currently underway to determine the effect of selected species isolated in the studies presented here on the functioning of the genome of wheat host plants.

In this work, field experiments were carried out using two cultivation systems—conventional and no_till type. In the conventional system, depending on the form of wheat, mineral fertilization was applied in autumn and in spring—directly after the beginning of the growing season. In addition, herbicide was applied. Meanwhile, no intervention was undertaken in no_till cultivation. The plots of both cultivation systems were separated by 50 m, but they were characterized by the same soil type, where the pre-crop was *Lathyrus sativus*. This may explain the existence of statistically insignificant differences in the composition of endogenous fungi of plants harvested from a field under conventional and no_till management (Figs. 5a, 6, 7, 8a). It can therefore be concluded that the mycobiome of the wheat endosphere is more affected by the presence of microorganisms appearing in the soil and air than mineral fertilization and the use of herbicides. In the dataset used in this study, field conditions accounted for approximately 9% of the variability in endophytic fungal communities. A similar finding was made from a study by Latz et al.^14^ who documented that airborne and rhizosphere fungi shaped the phyllosphere and root microbiome, respectively, and this factor introduced a variability of 5.98%. Moreover, a limited impact of the management strategy on fungal communities associated with plants was noted by Gdanetz and Trail^17^ and Hartman et al.^16^ On the other hand, in this study, the optimal growing conditions of wheat plants in greenhouse cultivation, with limited access of microorganisms from the air and soil, which was double autoclaved, favored the equal spread in the plants tissues of fungi from seeds. Interestingly, in the presented study, there were significant differences in the average number of ITS copies between plant samples from greenhouse (~ 1359), conventional (~ 23,530) and no_till (~ 29,660) conditions. This most likely suggests the existence of differences in the degree of colonization of the internal parts of wheat plants by fungi. It is presumed that there is a link between external conditions and endogenous fungal occupancy levels. Field conditions are much more demanding and more variable for plants than a greenhouse. It is worth emphasizing that although temperatures and precipitation favored the sowing of winter wheat in October 2018, when they amounted to 10 °C and 27 mm, respectively, and spring wheat in March 2019, where they amounted to 5.82 °C and 33 mm, respectively, June 2019 was warm but not very humid, while in July 2019, the temperature was only 19.83 °C, and the rainfall reached as much as 43 mm. Since each species of fungus may have different optimal temperature and humidity conditions needed for development, weather conditions during the entire vegetation cycle or at a specific stage of wheat development could of course determine not only the composition, but also the degree of colonization of the plant by the studied microorganisms. It seems, therefore, that the increased number of ITS copies registered in field plant samples may indicate that less favorable weather conditions during the plant vegetation period (April, May—dry and warm June, cold and rainy July) could increase the degree of colonization of plant tissues, which could become a kind of "waiting room" for fungi. However, in relation to the field conditions themselves, plants growing in no_till conditions showed a higher average number of ITS copies in their endosphere than those growing in conventional conditions where mineral fertilization and herbicides were used. According to the study by Karlsson and coworkers^56^ on plant epiphytes, plant protection products and mineral fertilization have a negative impact on the entire plant endosphere. This probably explains the resulting difference in the degree of plant colonization with endophytic fungi in conventional and no_till type cultivation, expressed here in numbers of ITS copies. Meanwhile, it was found that no_till cultivation without any treatments contributed to a greater colonization of wheat plants by fungi, mainly pathogenic ones, but with a simultaneous reduction in the diversity of fungi. Although there were no significant differences in alpha diversity ratios between the two groups of plants in the field, culture-dependent analyzes showed that plants grown in the no_till system had lower endophytic fungal diversity and contained fewer unique species than those grown in conventional plots (Fig. 8a). In addition, the observed lower biodiversity of fungal species in the endosphere of plants grown in no_till field experiments than those grown in conventional system could be due to the presence of *Puccinia* sp. (as shown in Fig. 5) in field environment. It is believed that this biotrophic pathogen dominated the ecological niche, especially those wheat plants that were cultivated without the addition of minerals and without the use of herbicides. This study also noted the existence of differences in the structures of endophytic fungal communities between plants grown in the field and in the greenhouse. It was documented that the diversity and species richness of fungi colonizing the internal tissues of wheat plants from greenhouses was greater than in field condition (Figs. 1a, 5a, 6). The plants harvested from the field were dominated by several species of fungi, which may indicate their competitiveness and ability to explore the wheat endosphere in natural, though less favorable for plant growth conditions. Previous reports show that the cultivation system influences the microbial composition more than its richness^16^, which is in line with this study’s findings (Figs. 5a, 8a). The results of current study indicate that the type of plant organ strongly influenced the wheat mycobiome with an effect of about 25%, which is also consistent with the previously available data^11,14,17^. The endogenous fungal diversity in leaves was found to be lower than in kernels, stems, and roots (Fig. 1b), which may correlate with the *Puccinia* sp. infection in field plants. Like previous studies^14^, it was found that the species richness and unique fungal species (Fig. 8b) in roots are significantly higher than in other wheat tissues (Fig. 1b). Furthermore, the above- and below-ground parts of the wheat plant present distinct structures of the endogenous fungal community (Fig. 8b). In addition, mycobiome analyses of 10 wheat cultivars revealed that the host genotype does not have an impact on the endophyte’s composition in the whole dataset, as well as separately in leaves, roots, stems, and kernels. Similar results were obtained by Comby et al.^11^. Contrarily, Latz et al.^14^ observed that the host genotype shapes the fungal endophytic structure in wheat leaves and roots. However, the mycobiome of roots and leaves sampled at anthesis (BBCH 65) from 12 related winter wheat cultivars with diverse resistance to Septoria tritici blotch (STB) was evaluated in the study. This varied sensitivity for the fungal pathogen or the different growth stages of analyzed plants (BBCH 77 in this study) is likely reflected in the endogenous fungal community and is the reason for different observations than here. Moreover, according to Zheng et al.^12^, who observed that the plant growth conditions (environment) had a stronger effect than the variety, the effect of host genotype may have been undetectable in the experimental setup of this study. In addition to the above, it was observed in this study that the wheat forms (winter and spring) also had no effect on endosphere differentiation. It can therefore be assumed that the vegetation period/length of these plants is not as important for the structure of the mycobiome as, for example, the growth conditions.

The present study analyzed *Triticum aestivum* plants from 10 cultivars, 3 management strategies, 4 plant organs, and 2 wheat forms. In all the studied conditions, one of the main groups inhabiting the wheat endosphere was the *Dothideomycetes* class members, and this is consistent with previous reports^11,17,20^. This study identified the *Cladosporium*, *Aureobasidium*, and *Alternaria* fungi among this class. Cultivar Arabella demonstrated the higher and cultivar Rusałka the lower level of *Dothideomycetes* in relative abundance across the wheat varieties. Additionally, these class members colonize mainly the roots and kernels. Interestingly, there was also an inverse relationship between the abundance of *Dothideomycetes* and *Pucciniomycetes* in the endosphere of the tested wheat cultivars (Fig. 5d). *Dothideomycetes* were observed less frequently when *Pucciniomycetes* (mainly members of the pathogenic Puccinia sp.) colonized the plant, suggesting competition between these fungi. Similarly, Rojas and coworkers^20^ observed that *Dothideomycetes* fungi (*Cladosporium*) negatively correlated with another wheat pathogen—*Fusarium graminearum,* in wheat spikes. Thus, this class member may be considered as the biological control agent in wheat. Other substantive groups of fungi identified by the culture independent ITS metabarcoding are *Sordariomycetes* (mainly observed genera: *Colletotrichum*, *Trichoderma*, *Acremonium*, *Sarocladium*, *Fusarium*, *Microdochium*, *Chaetomium*, *Gaeumannomyces*) and *Eurotiomycetes* (mainly *Penicillium* and *Chrysosporium* genera) classes (Fig. 5). It is worth emphasizing that the above genera were also identified by culture-dependent methods. The results showed that fungi from *Cladosporium*, *Penicillium*, *Sarocladium*, and *Fusarium* genera were present in all the studied conditions and plant organs and represented the core wheat microbiome. *Cladosporium* sp. were also observed in the endosphere of *Triticum aestivum* collected in France^11^, Canada^23^, Argentina^22^, Denmark, and Sweden^20^. Wheat endogenous representatives of *Penicillium* and *Sarocladium* family were reported in France^11^ and Canada^23^. Meanwhile, *Fusarium* sp. has been commonly observed in the endosphere of wheat grown in different, often disparate, geographical regions, such as in Poland^58^, North China^8^, or South Africa^59^. Species that were frequently isolated from wheat plants in this study and by other authors^8,21,60^ were *S. strictum, P. olsonii*, *F. proliferatum,* and *C. cladosporoides*.

To summarize, comprehensive analyses were performed on the diversity and community structures of wheat microbiome with simultaneous isolation of endophytic fungi, resulting the candidate strains for future analyses. The wheat microbiome is mainly altered by the type of plant organ and growth conditions, and management strategy affects the fungal community structure more than the fungal abundance. The effects of host genotype and wheat forms on wheat endosphere can be undetectable. *Dothideomycetes*, *Sordariomycetes*, and *Eurotiomycetes* class members dominate in the wheat endosphere. The core wheat microbiome consists of fungi of the *Cladosporium, Penicillium,* and *Sarocladium* genera. Further, the representatives of *Fusarium* and *Alternaria* genera were isolated from all tissue types of the wheat plants cultivated on the field. Fungi considered pathogenic coexist in the wheat endosphere with fungi known to be beneficial to plants. Wheat plants are a valuable source of potential biological control and growth-promoting agents.

## Material and methods

### Plant material and experimental design

Ten bread wheat cultivars: 5 winter forms (Legenda, Bamberka, Figura, Ostroga, Arkadia) and 5 spring forms (Rusałka, Rospuda, Bombona, Arabella and Kandela) were used in the presented study. These cultivars are common in Poland and considered to be moderately resistant to fungal diseases according to the agronomic assessments made by the owner companies (PHR Poznan Plant Breeding; HR Strzelce; DANKO). Wheat plants were grown in three groups with different growth conditions: conventional with mineral fertilization and herbicides, but no fungicides; “no-till” type without mineral fertilization, herbicides and fungicides in the field; under controlled conditions in the greenhouse with mineral fertilization.

### Field experiment

The field experiment was carried out in 2018/2019, on the IPG PAS experimental plots (GPS coordinates: N 52.521012, E 16.692005) characterized by poor sandy-clay soil. Plants were grown from field-collected seeds, directly received from the breeding stations on 1 m^2^ plots for every genotype. In both conventional and no-till plots, the pre-crop (2017, 2018) was grass pea (*Lathyrus sativus*). In autumn, on plots of conventional winter wheat cultivation, fertilization was applied in an amount ensuring 20 kg N/ha, 60 kg P/ha and 120 kg K/ha (Polifoska 5 (NPK(MgS) 5-15-30-(2-7), Grupa Azoty Zakłady Chemiczne "Police" S.A., Police, Poland). In spring, on plots of conventional winter and spring wheat cultivation, nitrate fertilizer was applied in an amount ensuring 70 kg N/ha (Grupa Azoty Zakłady Azotowe “Puławy” S.A., Puławy, Poland). Weeds were controlled with Legato 500SC (ADAMA Polska Sp. z oo, Warsaw) herbicide at a dose of 1.5 l/ha. The experiment was set up in a completely randomized design: two land management strategies (conventional and no-till), ten crop cultivars, three plots/replicates and three hundred plants per plot/replication.

The sowing date for winter wheat was in the first week of October 2018, and for spring wheat in the third week of March 2019. In the months from October 2018 to July 2019, the average temperature and precipitation according to data from meteorological station in the experimental field of the Institute of Plant Genetics of the Polish Academy of Sciences in Cerekwica were as follows: October—10 °C, 27 mm; November—5.22 °C, 10.6 mm; December—2.69 °C, 57.4 mm; January—1.83 °C, 47 mm; February—3.26 °C, 12 mm; March—5.82 °C, 33 mm; April—7.16 °C, 28.4 mm; May—15.67 °C, 58 mm; June—22.77 °C, 3.6 mm; July—19.83 °C and 43 mm.

### Greenhouse experiment

In the greenhouse experiment, plants were grown under controlled conditions in twice autoclaved sandy-clay soil mixed with quartz sand. Seeds obtained directly from breeding companies were sterilized by soaking in 70% ethyl alcohol (Avanator Performance Materials Poland S.A.—formerly: POCH S.A., Gliwice, Poland) for 30 s, and then in a solution prepared by mixing sodium hypochlorite (ACE Classic—Procter and Gamble DS. Poland Sp. z o. o., Warsaw, Poland) with distilled water in a ratio of 1:9 for 120 s. The seeds were then washed several times with distilled water to remove residual alcohol and sodium hypochlorite. Seeds were placed in sterile conditions on Petri glasses with a diameter of 15 cm lined with paper soaked in distilled water. After 3 days of incubation at room temperature, seedlings with similar lengths of roots and leaves were placed in double-walled pots with a diameter of 25 cm and a height of 35 cm filled with sterilized soil. The plants were fertilized with Polifoska 5 (NPK(MgS) 5-15-30-(2-7) (Grupa Azoty Zakłady Chemiczne "Police" S.A., Police, Poland) in the amount calculated as in the field experiment and were grown in a greenhouse under controlled conditions: photoperiod 12/12 h (day/night,) temperature 25 ± 3 °C. Humidity was maintained at 40% during light and dark periods. After 14 days, seedlings of winter wheat cultivars were vernalized at 4 °C for 8 weeks and photoperiod 16/8 h (day/night). After this time, all seedlings (spring and winter varieties) were grown at a temperature of 22/18 °C (day/night) and a photoperiod of 16/8 h (day/night). The greenhouse experiment was organized in a completely randomized system: 10 cultivars x three pot replicates x five plants per replicate.

### Sample collection and processing

**Figure 9:**
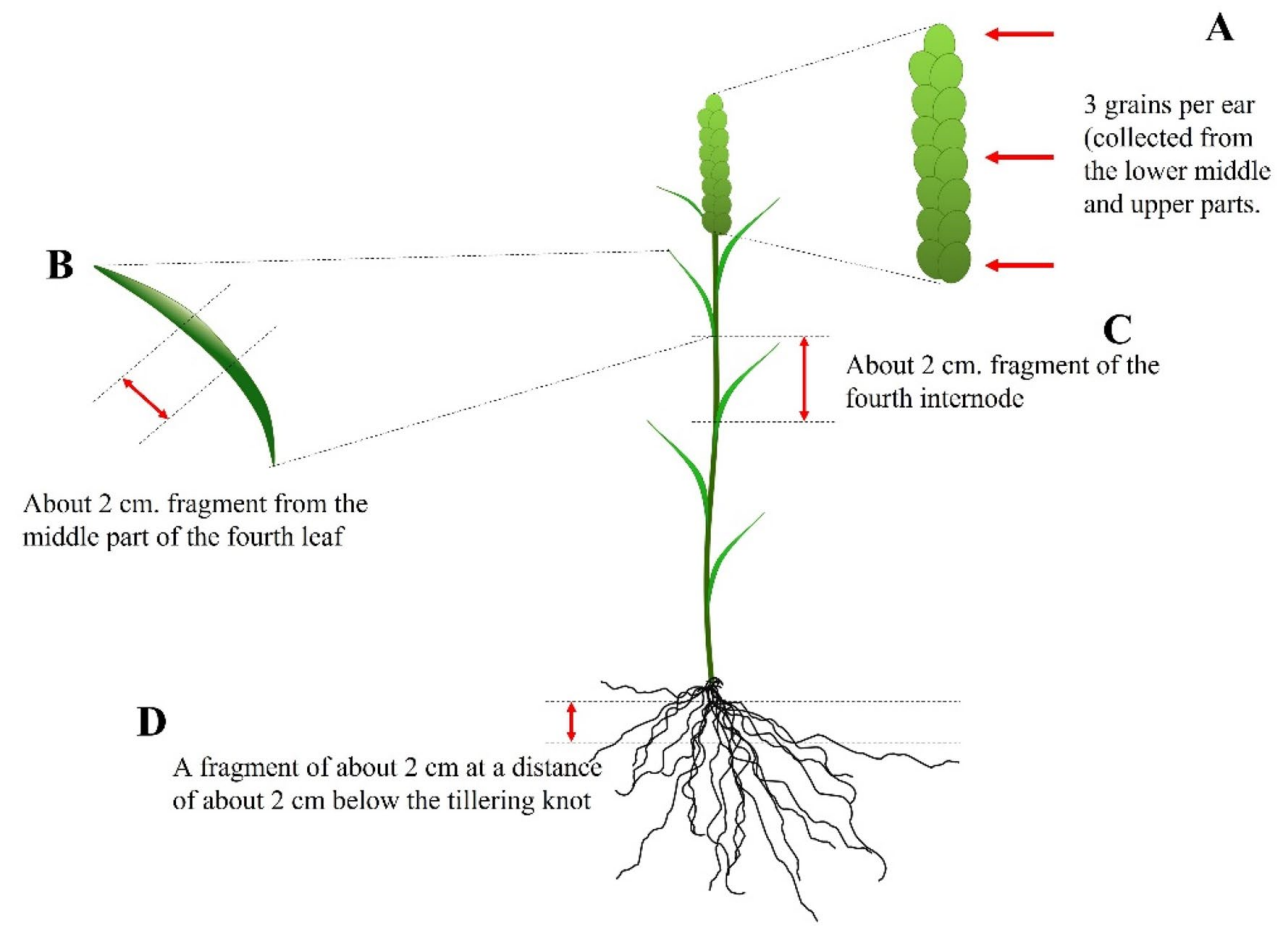
Material collection workflow.

Fresh plant samples (1 sample: 5 plants/tissue/wheat cultivars/land management strategies) were collected at the late milk stage (BBCH 77). Plants grown in greenhouse conditions and in field conditions were subjected to preliminary preparation. The surface of the roots was cleaned mechanically with a brush from the remaining soil. For the analysis, 3 ear kernels, a fragment from the middle part of the fourth leaf, a fragment of the fourth internode, and a fragment of roots approximately 2 cm below the tillering knot were taken. The individual parts of the plant (roots, stem, leaves) were then cut into approximately 2 cm long sections using a sterile scalpel and sterilized (Fig. 9). The seed coat was removed from the grains before they were sterilized. Plant fragments were rinsed in 70% ethyl alcohol (Avanator Performance Materials Poland S.A.) for 30 s, then in 0.5% sodium hypochlorite (ACE Classic—Procter and Gamble DS. Polska Sp.z o.o., Poland) for 2 min. The purified fragments were washed several times with distilled water to remove the residual ethyl alcohol and sodium hypochlorite.

### Identification of endogenous fungi using culture independent method

#### DNA extraction

The sterilized samples were lyophilized in 2 ml tubes. Next, the 40 mg of lyophilized tissue were homogenized with the use of stainless-steel beads (3 mm diameter) in Tissue Lyser MM400 (Retsch, Poland) for 2 min at 26 Hz. DNA isolation was performed using Wizard Genomic DNA Purification Kit (Promega, Madison, Wisconsin, United States), according to manufacturer instruction. Determination of quality and quantity of obtained DNA was conducted on Nanodrop spectrophotometer (ThermoFisher Scientific, Waltham, Massachusetts, USA).

#### Library preparation and sequencing

Library preparation was performed by amplification of ITS2 region using primer set described by White et al.^61^ with Illumina's adapters attached (ITS3: 5′-TCG TCG GCA GCG TCA GAT GTG TAT AAG AGA CAG GCA TCG ATG AAG AAC GCA GC-3′; ITS4R: 5′-GTC TCG TGG GCT CGG AGA TGT GTA TAA GAG ACA GTC CTC CGC TTA TTG ATA TGC-3′). PCR reactions were conducted with the use of Phusion Hot Start II High-Fidelity DNA Polymerase (Thermo Fisher Scientific, Waltham, Massachusetts, United States) and 100 ng of total DNA in T-1000 Thermal Cycler (BioRad, Hercules, California, United States). Reaction conditions were as follows: initial denaturation at 98 °C for 30 s, followed by 35 cycles of denaturation at 98 °C for 10 s, annealing at 55 °C for 20 s and elongation at 72 °C for 15 s, and at the end 10 min elongation step at 72 °C. Illumina MiSeq in 2 × 300 bp paired-end format was performed for ITS2 sequencing with the use of MiSeq Reagent Kit v3 (Illumina, California, United States). Second stage of library preparation, sequencing of 120 samples and sequence pre-processing was conducted in Genomed company (Warsaw, Poland).

### Identification of endogenous fungi using culture-dependent method

The sterilized sections of all plant parts and the kernels were placed on potato dextrose agar (PDA) (Oxoid Thermo Fisher Scientific, Waltham, MA, USA) with the addition of 50 µl of ampicillin (Merck Life Science Sp.z.o.o., Darmstadt, Germany) at a concentration of 100 mg/ml in 90 mm Petri dishes, under sterile conditions. The kernels were cut along the axial plane and placed with the inside of the kernel towards the substrate before being placed on the medium. The cultivation was carried out in an incubator (Sanyo MIR-254, SanLab, Poland) at the temperature of 21 °C and with photoperiodism 12/12 (day/night) until the growth of the fungal cultures was observed, which were then passaged several times into Petri dishes with a diameter of 90 mm with PDA medium in to obtain monocultures. The mycelium was collected in 1.5 ml Eppendorf tubes and stored at − 20 °C until DNA isolation.

The obtained fungal isolates were collected by inoculation under sterile conditions with PDA and SNA media solidified in 2 ml cryogenic tubes. Two copies of the collection were created, one of which is stored at − 80 °C—for this purpose, after several days of incubation at 21 °C, a 10% solution of glycerol in water (v/v) was added to the cryogenic tubes, and the second copy of the fungi collection is kept at 4 °C. DNA extraction was performed with the use of Wizard Genomic DNA Purification Kit (Promega, Wisconsin, United States) from 40 mg of mycelium obtained from homogeneous cultures. For DNA barcoding of the obtained fungal strains, the internal transcribed spacer (ITS) region, small-subunit (SSU) or large-subunit (LSU) nrRNA, and protein-coding markers (beta-tubulin (*tub2*), actin (*act*) and translation elongation factor 1-alpha (*tef1*)) were analyzed. The amplification and sequencing of the selected DNA fragments were performed according to previous research^58^.

#### Data processing

Classification of the reads were performed with the use of QIIME based on reference sequence from UNITE v8 (version 18.11.2018) database^62,63^. Adaptors, as well as low quality sequences were removed (quality < 20, minimal length 30) with the use of cutadapt^64^. Using fastq-join algorithm the pair ends were merged^65^. The *uclust* and *usearch61* algorithms were used for sequence clustering based on selected reference database and for removing chimeric sequences, respectively^66^. The ITS2 reads were clustered at 97% similarity level. Taxonomy assignment for obtained sequences was performed based on UNITE database using *blast* algorithm^67^. Sequence classified as *Viridiplantae* and *Metazoa* kingdoms were removed for further analysis.

#### Diversity and statistical analysis

Bioinformatics as well as statistical analyses were conducted with the use of R version 3.6.3^68^, unless otherwise specified. The alpha diversity was calculated and visualized using plot_richness function in phyloseq^69^ version 1.30.0. The measured alpha diversity estimates were compared between studied groups with the used of and Wilcoxon Rank Sum test using the pairwise comparisons (pairwise.wilcox.test function). Weighted UniFrac distance calculations, principal coordinate analysis was conducted in phyloseq and microbiome v. 1.8.0.^70^ packages. To examine the effect of growth conditions, tissue types, wheat form and cultivar on endophytic fungi community structure in wheat, the adonis formula from *vegan* package were applied using a nonparametric permutational ANOVA multivariate (PERMANOVA) test. Permutation test for homogeneity of multivariate dispersions (variances) with the use of betadisper function in vegan package, were performed and compared using the permutest function. The visualization of the wheat endophytic fungal community structure (Fig. 5) was conducted with the use of phyloseq^69^ and ggplot2 v.3.3.5 packages^72^. The heat trees (Fig. 6) and heat map (Fig. 7) were performed with the use of MicrobiomeAnalyst version 2.0^74^ (https://new.microbiomeanalyst.ca//ModuleView.xhtml). The Linear Discriminant Analysis Effect Size (LEfSe) from MicrobiomeAnalyst^74^ was used to identify and visualize the condition- and tissue- associated OTUs (Fig. 4). The Fig. 9 was draw in Microsoft Office Power Point 2021 software.

### Statements

Experimental and field studies on crop plants, including collection and storage of plant material, were in accordance with the guidelines and legislation of the Institute of Plant Genetics of the Polish Academy of Sciences and Poland.

Breeding companies directly provided wheat seeds for experimental research, including field research, and granted permission to use the donated wheat seeds and the obtained plant progeny for further analyses.

## Supplementary Information

Supplementary Table 1.

## Data Availability

The datasets generated during the current study are available in the NCBI repository (BioProject PRJNA899455 and accession numbers: MW713455–MZ447578, OK328116–OK328169, OK358458–OK358468, OK337760–OK337828, OK652550 listed in Supplementary Table 1). Part of the sequence data that has been deposited in the NCBI repository is under verification (request ID: 2310230 2310260 2310272 2310335 2310339 2310341–2310343 2331590 2462038 2501709 2501725 2501727 2503041 2503045 2503067 2503076 2503079 2503097 2503106 2503424 2503438 2503443 2503734 2503748 2503751 2503757 2503767 2503768 2503773 2503776 2505790 2505791 2505793–2505795 2505797 2505798 2505855 2505857 2505858 2505860 2512305 2546702 2547108 2547113 2547163 2547219 2547236 2547255).
