# Author Correction: Constellation of the endophytic mycobiome in spring and winter wheat cultivars grown under various conditions

**DOI:** 10.1038/s41598-023-35261-x

**Published:** 2023-05-22

**Authors:** Sylwia Salamon, Katarzyna Mikołajczak, Lidia Błaszczyk

**Affiliations:** Department of Plant Microbiomics, Institute of Plant Genetics PAS, Poznan, Poland

Correction to: *Scientific Reports* 10.1038/s41598-023-33195-y, published online 13 April 2023

The original version of this Article contained an error in the name of the authors Sylwia Salamon, Katarzyna Mikołajczak and Lidia Błaszczyk which were incorrectly given as Salamon Sylwia, Mikołajczak Katarzyna and Błaszczyk Lidia.

The original Article has been corrected.

