# Supplementary material for: Constellation of the endophytic mycobiome in spring and winter wheat cultivars grown under various conditions
Source: Sci Rep. 2023 Apr 13;13:6089. doi: 10.1038/s41598-023-33195-y (PMC10102161; doi:10.1038/s41598-023-33195-y)
Supplement: Supplementary file 1 — Supplementary Table 1. [file 41598_2023_33195_MOESM1_ESM.docx]

**Table S1** List of isolated wheat’s endophytic fungi and GenBank accession numbers of sequences used for identification.

* - Sequence deposited, awaiting assignment of accession number.

| **ID** | **Form** | **Cultivar** | **Tissue** | **Conditions** | **Species/**  **genera** | **GenBank accession number** | | | | |  |  |
| --- | --- | --- | --- | --- | --- | --- | --- | --- | --- | --- | --- | --- |
|  |  |  |  |  |  | ITS | LROR | NS | *btub* | *ef1* | *act (act1f)* | *cmd* |
| E1 | spring wheat | Rospuda | leaf | greenhouse | *Sarocladium spinificis* | MW713455 | OK337760 |  |  |  | OK358458 |  |
| E2 | spring wheat | Rospuda | leaf | greenhouse | *Sarocladium spinificis* | MW713456 | OK337761 |  |  |  | OK358459 |  |
| E3 | spring wheat | Rospuda | stem | greenhouse | *Sarocladium spinificis* | MW713457 | OK337762 |  |  |  | OK358460 |  |
| E4 | spring wheat | Rospuda | stem | greenhouse | *Sarocladium spinificis* | MW713458 | OK337763 |  |  |  | OK358461 |  |
| E5 | spring wheat | Rospuda | seed | greenhouse | *Sarocladium spinificis* | MW713459 | OK337764 |  |  |  | OK358462 |  |
| E6 | spring wheat | Rospuda | seed | greenhouse | *Penicillium chrysogenum* | MW713460 |  |  |  |  |  | * |
| E7 | spring wheat | Rospuda | seed | greenhouse | *Sarocladium spinificis* | MW713461 | OK337765 |  |  |  | OK358463 |  |
| E8 | spring wheat | Rospuda | seed | greenhouse | *Sarocladium spinificis* | MW713462 | OK337766 |  |  |  |  |  |
| E11 | spring wheat | Rospuda | root | greenhouse | *Sarocadium spinificis* | MW713463 | OK337767 |  |  |  | OK358464 |  |
| E12 | spring wheat | Rospuda | root | greenhouse | *Fusarium sp.* | MW888889 |  |  |  |  |  |  |
| E13 | spring wheat | Rospuda | root | greenhouse | *Sarocadium spinificis* | MW713464 | OK337768 |  |  |  | OK358465 |  |
| E15 | spring wheat | Rospuda | root | greenhouse | *Sarocladium spinificis* | MW713465 | OK337769 |  |  |  |  |  |
| E16 | spring wheat | Rospuda | root | greenhouse | *Sarocladium spinificis* | MW713466 | OK337770 |  |  |  |  |  |
| E17 | spring wheat | Rospuda | root | greenhouse | *Sarocladium spinificis* | MW713467 | OK337771 |  |  |  | OK358466 |  |
| E18 | spring wheat | Rospuda | root | greenhouse | *Sarocladium spinificis* | MW713468 | OK337772 |  |  |  | OK358467 |  |
| E20 | spring wheat | Rusałka | leaf | greenhouse | *Penicillium chrysogenum* | MW713469 |  |  | * |  |  | * |
| E21 | spring wheat | Rusałka | stem | greenhouse | *Sarocladium strictum* | MZ447431 | OK337774 |  |  | * | OK358468 |  |
| E22 | spring wheat | Rusałka | stem | greenhouse | *Sarocladium strictum* | MZ447432 | OK337775 |  |  | * |  |  |
| E23 | spring wheat | Rusałka | seed | greenhouse | *Sarocladium strictum* | MZ447433 | OK337776 |  |  | * |  |  |
| E24 | spring wheat | Rusałka | seed | greenhouse | *Sarocladium strictum* | MZ447434 | OK337777 |  | * | * |  |  |
| E25 | spring wheat | Rusałka | seed | greenhouse | *Sarocladium strictum* | MZ447435 | OK337778 |  |  | * |  |  |
| E26 | spring wheat | Rusałka | seed | greenhouse | *Sarocladium strictum* | MZ447436 | OK337779 |  |  |  |  |  |
| E27 | spring wheat | Rusałka | seed | greenhouse | *Cladosporium sp.* | MZ447437 |  | OK328116 |  | * |  |  |
| E28 | spring wheat | Bombona | leaf | greenhouse | *Penicillium digitatum* | MZ447438 |  |  | * | * |  |  |
| E30 | spring wheat | Bombona | root | greenhouse | *Trichoderma sp.* |  |  |  |  | * |  |  |
| E31 | spring wheat | Bombona | root | greenhouse | *Penicilium sp.* |  |  |  |  | * |  |  |
| E32 | spring wheat | Bombona | seed | greenhouse | *Penicillium olsonii* | MZ447439 |  |  | * |  |  |  |
| E33 | spring wheat | Bombona | seed | greenhouse | *Penicillium olsonii* | MW888890 |  |  | * |  |  |  |
| E35 | spring wheat | Bombona | seed | greenhouse | *Engyodontium album* | MZ447440 |  | OK328117 |  |  |  |  |
| E36 | spring wheat | Bombona | seed | greenhouse | *Engyodontium album* | MZ447441 |  | OK328118 |  |  |  |  |
| E37 | spring wheat | Bombona | seed | greenhouse | *Engyodontium album* | MZ447442 |  | OK328119 |  |  |  |  |
| E38 | spring wheat | Bombona | seed | greenhouse | *Penicillium digitatum* | MZ447443 |  |  | * | * |  |  |
| E39 | spring wheat | Bombona | seed | greenhouse | *Penicillium olsonii* | MZ447444 |  |  | * |  |  |  |
| E40 | spring wheat | Kandela | leaf | greenhouse | *Penicillium digitatum* | MZ447445 |  |  | * | * |  |  |
| E41 | spring wheat | Kandela | leaf | greenhouse | *Sarocladium spinificis* | MZ447446 | OK337780 |  |  |  |  |  |
| E42 | spring wheat | Kandela | leaf | greenhouse | *Penicillium olsonii* |  |  |  | * |  |  |  |
| E43 | spring wheat | Kandela | leaf | greenhouse | *Penicillium olsonii* | MW888891 |  |  | * |  |  |  |
| E44 | spring wheat | Kandela | leaf | greenhouse | *Sarocladium spinificis* | MZ447447 | OK337781 |  |  | * | * |  |
| E45 | spring wheat | Kandela | leaf | greenhouse | *Acremonium sp.* | MZ447448 |  |  |  |  |  |  |
| E46 | spring wheat | Kandela | leaf | greenhouse | *Penicillium olsonii* |  |  |  | * |  |  |  |
| E47 | spring wheat | Kandela | leaf | greenhouse | *Sarocladium spinificis* | MW888892 | OK337782 |  |  |  | * |  |
| E48 | spring wheat | Kandela | leaf | greenhouse | *Sarocladium spinificis* | MZ447449 | OK337783 |  |  |  | * |  |
| E49 | spring wheat | Kandela | leaf | greenhouse | *Sarocladium spinificis* | MZ447450 | OK337784 |  |  |  | * |  |
| E51 | spring wheat | Kandela | stem | greenhouse | *Penicillium olsonii* | MZ447451 |  |  | * |  |  |  |
| E52 | spring wheat | Kandela | stem | greenhouse | *Penicillium olsonii* | MZ447452 |  |  | * |  |  |  |
| E54 | spring wheat | Kandela | stem | greenhouse | *Sarocladium spinificis* | MZ447453 | OK337786 |  |  |  |  |  |
| E55 | spring wheat | Kandela | stem | greenhouse | *Sarocladium sp.* | MZ447454 |  |  |  |  |  |  |
| E56 | spring wheat | Kandela | stem | greenhouse | *Penicillium olsonii* | MZ447455 |  |  | * |  |  |  |
| E57 | spring wheat | Kandela | stem | greenhouse | *Sarocadium spinificis* | MZ447456 | OK337787 |  |  |  |  |  |
| E58 | spring wheat | Kandela | stem | greenhouse | *Penicilium sp.* |  |  |  |  | * |  | OK652550 |
| E59 | spring wheat | Kandela | stem | greenhouse | *Penicilium sp.* | MZ447457 |  |  |  |  |  |  |
| E60 | spring wheat | Kandela | stem | greenhouse | *Penicilium sp.* | MZ447458 |  |  | * |  |  |  |
| E62 | spring wheat | Kandela | stem | greenhouse | *Phlebia sp.* | MZ447459 |  |  |  |  |  |  |
| E63 | spring wheat | Kandela | root | greenhouse | *Penicillium olsonii* | MZ447460 |  |  | * |  |  |  |
| E65 | spring wheat | Kandela | root | greenhouse | *Sarocladium spinificis* | MZ447461 | OK337788 |  |  |  | * |  |
| E66 | spring wheat | Kandela | root | greenhouse | *Penicillium digitatum* | MW888893 |  |  | * |  |  |  |
| E67 | spring wheat | Kandela | root | greenhouse | *Penicillium olsonii* | MZ447462 |  |  |  |  |  |  |
| E68 | spring wheat | Kandela | root | greenhouse | *Penicillium olsonii* | MZ447463 |  |  | * |  |  |  |
| E69 | spring wheat | Kandela | seed | greenhouse | *Acremonium sclerotigenum* | MZ447464 |  |  | * |  |  |  |
| E70 | spring wheat | Kandela | seed | greenhouse | *Penicillium olsonii* | MW888894 |  |  | * |  |  |  |
| E71 | spring wheat | Kandela | seed | greenhouse | *Penicillium olsonii* |  |  |  | * |  |  |  |
| E72 | spring wheat | Kandela | seed | greenhouse | *Lecanicillium lecanii* |  |  |  |  |  |  |  |
| E73 | spring wheat | Kandela | seed | greenhouse | *Sarocladium sp.* | MZ447465 |  |  |  |  |  |  |
| E74 | spring wheat | Bombona | seed | greenhouse | *Engyodontium album* | MZ447466 |  | OK328120 |  |  |  |  |
| E75 | spring wheat | Kandela | stem | greenhouse | *Penicilium sp.* | MZ447467 |  |  |  |  |  |  |
| E77 | spring wheat | Arabella | leaf | greenhouse | *Penicillium olsonii* | MZ447468 |  |  | * |  |  |  |
| E78 | spring wheat | Arabella | stem | greenhouse | *Penicillium olsonii* | MZ447469 |  |  | * |  |  |  |
| E79 | spring wheat | Arabella | stem | greenhouse | *Sarocladium spinificis* | MZ447470 | OK337789 |  |  |  |  |  |
| E80 | spring wheat | Arabella | root | greenhouse | *Trichoderma viride* | MZ447471 |  |  |  | * |  |  |
| E81 | spring wheat | Arabella | root | greenhouse | *Trichoderma hamatum* | MZ447472 |  |  |  | * |  |  |
| E82 | spring wheat | Arabella | seed | greenhouse | *Acremonium sclerotigenum* | MZ447473 |  |  | * |  |  |  |
| E83 | spring wheat | Arabella | seed | greenhouse | *Clonostachys candelabrum* | MZ447474 | OK337790 |  | * |  |  |  |
| E84 | spring wheat | Arabella | seed | greenhouse | *Penicillium olsonii* | MZ447475 |  |  | * |  |  |  |
| E85 | spring wheat | Arabella | seed | greenhouse | *Penicillium olsonii* | MW888895 |  |  | * |  |  |  |
| E86 | winter wheat | Bamberka | seed | greenhouse | *Sarocladium spinificis* | MZ447476 | OK337791 |  |  |  |  |  |
| E87 | winter wheat | Bamberka | seed | greenhouse | *Sarocladium spinificis* | MZ447477 | OK337792 |  |  |  |  |  |
| E88 | winter wheat | Bamberka | seed | greenhouse | *Penicilium sp.* | MZ447478 |  |  |  |  |  |  |
| E89 | winter wheat | Bamberka | seed | greenhouse | *Sarocladium spinificis* | MZ447479 | OK337793 |  |  |  |  |  |
| E90 | winter wheat | Bamberka | seed | greenhouse | *Sarocladium spinificis* | MZ447480 | * |  |  |  |  |  |
| E91 | winter wheat | Bamberka | seed | greenhouse | *Sarocladium sp.* | MZ447481 |  |  |  |  |  |  |
| E92 | winter wheat | Bamberka | seed | greenhouse | *Sarocladium spinificis* | MZ447482 | OK337794 |  |  |  |  |  |
| E93 | winter wheat | Bamberka | seed | greenhouse | *Sarocladium spinificis* | MZ447483 | * |  |  |  |  |  |
| E94 | winter wheat | Bamberka | seed | greenhouse | *Sarocladium spinificis* | MZ447484 | OK337795 |  |  |  |  |  |
| E95 | winter wheat | Bamberka | leaf | greenhouse | *Sarocladium spinificis* | MZ447485 | * |  |  |  |  |  |
| E96 | winter wheat | Bamberka | leaf | greenhouse | *Sarocladium spinificis* | MZ447486 | OK337796 |  |  |  |  |  |
| E97 | winter wheat | Bamberka | leaf | greenhouse | *Cladosporium cladosporioides* | MZ447487 |  | OK328121 |  | * |  |  |
| E99 | winter wheat | Bamberka | leaf | greenhouse | *Cladosporium cladosporioides* | MW888896 |  | OK328122 |  | * |  |  |
| E101 | winter wheat | Bamberka | leaf | greenhouse | *Sarocladium spinificis* | MZ447488 | OK337797 |  |  |  |  |  |
| E102 | winter wheat | Bamberka | leaf | greenhouse | *Sarocladium spinificis* | MZ447489 | OK337798 |  |  |  |  |  |
| E103 | winter wheat | Bamberka | leaf | greenhouse | *Penicillium crustosum* | MZ447490 |  |  | * |  |  |  |
| E104 | winter wheat | Bamberka | leaf | greenhouse | *Sarocladium spinificis* | MZ447491 | OK337799 |  |  |  |  |  |
| E105 | winter wheat | Bamberka | leaf | greenhouse | *Sarocladium spinificis* | MZ447492 | OK337800 |  |  |  |  |  |
| E106 | winter wheat | Bamberka | leaf | greenhouse | *Penicillium crustosum* | MZ447493 |  |  | * |  |  |  |
| E108 | winter wheat | Bamberka | leaf | greenhouse | *Sarocladium spinificis* | MZ447494 | OK337801 |  |  |  |  |  |
| E109 | winter wheat | Bamberka | stem | greenhouse | *Sarocladium spinificis* | MZ447495 | OK337802 |  |  |  |  |  |
| E110 | winter wheat | Bamberka | stem | greenhouse | *Penicillium crustosum* | MZ447496 |  |  | * |  |  |  |
| E111 | winter wheat | Bamberka | stem | greenhouse | *Penicillium crustosum* | MW888897 |  |  | * |  |  |  |
| E112 | winter wheat | Bamberka | stem | greenhouse | *Penicillium crustosum* | MZ447497 |  |  | * |  |  |  |
| E113 | winter wheat | Bamberka | stem | greenhouse | *Penicillium crustosum* |  |  |  | * |  |  |  |
| E114 | winter wheat | Bamberka | stem | greenhouse | *Geomyces pannorum* | MZ447498 |  |  | * |  |  |  |
| E115 | winter wheat | Bamberka | stem | greenhouse | *Geomyces pannorum* | MZ447499 |  |  | * |  |  |  |
| E116 | winter wheat | Bamberka | stem | greenhouse | *Penicillium olsonii* | MZ447500 |  |  | * |  |  |  |
| E117 | winter wheat | Bamberka | stem | greenhouse | *Sarocladium spinificis* | MZ447501 | * |  |  |  |  |  |
| E118 | winter wheat | Bamberka | stem | greenhouse | *Sarocladium sp.* | MZ447502 |  |  |  |  |  |  |
| E119 | winter wheat | Bamberka | root | greenhouse | *Fusarium proliferatum* | MZ447503 |  |  |  | * |  |  |
| E120 | winter wheat | Bamberka | root | greenhouse | *Fusarium proliferatum* | MZ447504 |  |  |  | * |  |  |
| E121 | winter wheat | Bamberka | root | greenhouse | *Fusarium proliferatum* | MZ447505 |  |  |  | * |  |  |
| E122 | winter wheat | Bamberka | root | greenhouse | *Fusarium proliferatum* | MZ447506 |  |  |  | * |  |  |
| E124 | winter wheat | Ostroga | seed | greenhouse | *Sarocladium spinificis* | MZ447507 | OK337803 |  |  |  |  |  |
| E125 | winter wheat | Ostroga | seed | greenhouse | *Penicillium crustosum* | MW888899 |  |  | * |  |  |  |
| E126 | winter wheat | Ostroga | seed | greenhouse | *Penicilium sp.* | MZ447508 |  |  |  |  |  |  |
| E127 | winter wheat | Ostroga | seed | greenhouse | *Aspergillus sp.* | MZ447509 |  |  | * |  |  |  |
| E128 | winter wheat | Ostroga | seed | greenhouse | *Sarocladium spinificis* | MZ447510 | OK337804 |  |  |  |  |  |
| E129 | winter wheat | Ostroga | seed | greenhouse | *Penicillium digitatum* | MZ447511 |  |  | * |  |  |  |
| E130 | winter wheat | Ostroga | seed | greenhouse | *Penicillium digitatum* | MZ447512 |  |  | * |  |  |  |
| E131 | winter wheat | Ostroga | seed | greenhouse | *Sarocladium spinificis* | MZ447513 | OK337805 |  |  |  |  |  |
| E132 | winter wheat | Ostroga | seed | greenhouse | *Sarocladium spinificis* | MZ447514 | OK337806 |  |  |  |  |  |
| E133 | winter wheat | Ostroga | leaf | greenhouse | *Fusarium sp.* | MZ447515 |  |  |  |  |  |  |
| E134 | winter wheat | Ostroga | leaf | greenhouse | *Fusarium proliferatum* | MZ447516 |  |  |  | * |  |  |
| E135 | winter wheat | Ostroga | leaf | greenhouse | *Fusarium proliferatum* | MZ447517 |  |  |  | * |  |  |
| E136 | winter wheat | Ostroga | leaf | greenhouse | *Sarocladium spinificis* | MZ447518 | OK337807 |  |  |  |  |  |
| E137 | winter wheat | Ostroga | leaf | greenhouse | *Sarocladium sp.* | MZ447519 |  |  |  |  |  |  |
| E138 | winter wheat | Ostroga | leaf | greenhouse | *Sarocladium spinificis* | MZ447520 | OK337808 |  |  |  |  |  |
| E139 | winter wheat | Ostroga | leaf | greenhouse | *Sarocladium spinificis* | MZ447521 | OK337809 |  |  |  |  |  |
| E140 | winter wheat | Ostroga | stem | greenhouse | *Sarocladium spinificis* | MZ447522 | OK337810 |  |  |  |  |  |
| E141 | winter wheat | Ostroga | stem | greenhouse | *Sarocladium spinificis* | MZ447523 | OK337811 |  |  |  |  |  |
| E142 | winter wheat | Ostroga | stem | greenhouse | *Sarocladium spinificis* | MZ447524 | OK337812 |  |  |  |  |  |
| E143 | winter wheat | Ostroga | stem | greenhouse | *Sarocladium spinificis* | MZ447525 | OK337813 |  |  |  |  |  |
| E146 | winter wheat | Ostroga | stem | greenhouse | *Penicillium crustosum* | MZ447526 |  |  | * |  |  |  |
| E147 | winter wheat | Ostroga | stem | greenhouse | *Penicillium crustosum* | MZ447527 |  |  | * |  |  |  |
| E148 | winter wheat | Ostroga | stem | greenhouse | *Sarocladium spinificis* | MZ447528 | OK337814 |  |  |  |  |  |
| E149 | winter wheat | Ostroga | stem | greenhouse | *Sarocladium spinificis* | MZ447529 | OK337815 |  |  |  |  |  |
| E150 | winter wheat | Ostroga | root | greenhouse | *Sarocladium spinificis* | MZ447530 | * |  |  |  |  |  |
| E151 | winter wheat | Ostroga | root | greenhouse | *Sarocladium spinificis* | MZ447531 | OK337816 |  |  |  |  |  |
| E153 | winter wheat | Ostroga | root | greenhouse | *Sarocladium sp.* | MZ447533 |  |  |  |  |  |  |
| E154 | winter wheat | Arkadia | leaf | greenhouse | *Acremonium sclerotigenum* | MZ447534 |  |  | * |  |  |  |
| E155 | winter wheat | Arkadia | leaf | greenhouse | *Cladosporium cladosporioides* | MZ447535 |  | OK328123 |  |  | * |  |
| E156 | winter wheat | Arkadia | stem | greenhouse | *Sarocladium spinificis* | MZ447536 | OK337817 |  |  |  |  |  |
| E157 | winter wheat | Arkadia | stem | greenhouse | *Sarocladium spinificis* | MZ447537 | OK337818 |  |  |  |  |  |
| E160 | winter wheat | Arkadia | stem | greenhouse | *Sarocladium spinificis* | MZ447538 | OK337819 |  |  |  |  |  |
| E161 | winter wheat | Arkadia | stem | greenhouse | *Chrysosporium pseudomerdarium* | MZ447539 |  |  |  |  | * |  |
| E162 | winter wheat | Arkadia | stem | greenhouse | *Chrysosporium pseudomerdarium* | MZ447540 |  |  | * |  | * |  |
| E164 | winter wheat | Arkadia | root | greenhouse | *Sarocladium spinificis* | MZ447541 | OK337820 |  |  |  |  |  |
| E165 | winter wheat | Arkadia | root | greenhouse | *Sarocladium sp.* | MZ447542 |  |  |  |  |  |  |
| E166 | winter wheat | Arkadia | root | greenhouse | *Fusarium proliferatum* | MZ447543 |  |  |  | * |  |  |
| E167 | winter wheat | Legenda | seed | greenhouse | *Sarocladium spinificis* | MZ447544 | OK337821 |  |  |  |  |  |
| E168 | winter wheat | Legenda | seed | greenhouse | *Sarocladium sp.* | MZ447545 |  |  |  |  |  |  |
| E169 | winter wheat | Legenda | leaf | greenhouse | *Sarocladium spinificis* | MZ447546 | OK337822 |  |  |  |  |  |
| E170 | winter wheat | Legenda | leaf | greenhouse | *Sarocladium spinificis* | MZ447547 | OK337823 |  |  |  |  |  |
| E171 | winter wheat | Legenda | leaf | greenhouse | *Sarocladium sp.* | MZ447548 | OK337824 |  |  |  |  |  |
| E172 | winter wheat | Legenda | leaf | greenhouse | *Sarocladium sp.* | MZ447549 |  |  |  |  |  |  |
| E173 | winter wheat | Legenda | leaf | greenhouse | *Sarocladium spinificis* | MZ447550 | OK337825 |  |  |  |  |  |
| E175 | winter wheat | Legenda | stem | greenhouse | *Sarocladium spinificis* | MZ447551 | OK337826 |  |  |  |  |  |
| E178 | winter wheat | Legenda | stem | greenhouse | *Penicillium crustosum* | MZ447552 |  |  | * |  |  |  |
| E179 | winter wheat | Legenda | stem | greenhouse | *Sarocladium spinificis* | MZ447553 | * |  |  |  |  |  |
| E181 | winter wheat | Legenda | stem | greenhouse | *Sarocladium sp.* | MZ447554 | OK337827 |  |  |  |  |  |
| E183 | winter wheat | Legenda | root | greenhouse | *Fusarium proliferatum* | MZ447555 |  |  |  | * |  |  |
| E184 | winter wheat | Legenda | root | greenhouse | *Penicilium sp.* |  |  |  |  |  |  |  |
| E186 | winter wheat | Legenda | root | greenhouse | *Acremonium sclerotigenum* |  |  |  | * | * |  |  |
| E187 | winter wheat | Legenda | root | greenhouse | *Fusarium proliferatum* | MZ447556 |  |  |  | * |  |  |
| E188 | winter wheat | Legenda | root | greenhouse | *Fusarium proliferatum* | MZ447557 |  |  |  | * |  |  |
| E189 | winter wheat | Legenda | root | greenhouse | *Sarocladium sp.* | MZ447558 |  |  |  |  |  |  |
| E190 | winter wheat | Legenda | root | greenhouse | *Sarocladium spinificis* | MZ447559 | OK337828 |  |  |  |  |  |
| E191 | winter wheat | Euforia | seed | greenhouse | *Trichoderma koningii* | MZ447560 |  |  |  |  |  |  |
| E192 | winter wheat | Euforia | seed | greenhouse | *Trichoderma sp.* |  |  |  |  |  |  |  |
| E193 | winter wheat | Euforia | seed | greenhouse | *Penicillium expansum* | MZ447561 |  |  | * |  |  |  |
| E194 | winter wheat | Euforia | seed | greenhouse | *Sarocladium sp.* | MW888900 |  |  |  |  |  |  |
| E196 | winter wheat | Euforia | seed | greenhouse | *Penicillium olsonii* | MZ447562 |  |  | * |  |  |  |
| E197 | winter wheat | Euforia | seed | greenhouse | *Trichoderma viride* | MZ447563 |  |  |  |  |  |  |
| E199 | winter wheat | Euforia | seed | greenhouse | *Sarocladium sp.* | OM478508 |  |  |  |  |  |  |
| E200 | winter wheat | Euforia | seed | greenhouse | *Fusarium proliferatum* | MZ447564 |  |  |  | * |  |  |
| E201 | winter wheat | Euforia | seed | greenhouse | *Fusarium proliferatum* | MZ447565 |  |  |  | * |  |  |
| E202 | winter wheat | Euforia | seed | greenhouse | *Fusarium proliferatum* | OM478509 |  |  |  | * |  |  |
| E203 | winter wheat | Euforia | seed | greenhouse | *Sarocladium sp.* | MZ447566 |  |  |  |  |  |  |
| E206 | winter wheat | Euforia | leaf | greenhouse | *Aspergillus sp.* | MZ447568 |  |  |  |  |  |  |
| E207 | winter wheat | Euforia | leaf | greenhouse | *Sarocladium sp.* | MZ447569 |  |  |  |  |  |  |
| E208 | winter wheat | Euforia | leaf | greenhouse | *Nigrospora gorlenkoana* | MZ447570 |  |  | * |  |  |  |
| E209 | winter wheat | Euforia | leaf | greenhouse | *Sarocladium sp.* | MZ447571 |  |  |  |  |  |  |
| E210 | winter wheat | Euforia | stem | greenhouse | *Penicilium sp.* | MZ447572 |  |  |  |  |  |  |
| E213 | winter wheat | Euforia | stem | greenhouse | *Sarocladium sp.* | MW888901 |  |  |  |  |  |  |
| E215 | winter wheat | Euforia | stem | greenhouse | *Sarocladium sp.* | MW888902 |  |  |  |  |  |  |
| E218 | winter wheat | Euforia | stem | greenhouse | *Penicilium sp.* |  |  |  |  |  |  |  |
| E219 | winter wheat | Euforia | stem | greenhouse | *Fusarium proliferatum* | MZ447573 |  |  |  | * |  |  |
| E220 | winter wheat | Euforia | root | greenhouse | *Sarocladium sp.* | MZ447574 |  |  |  |  |  |  |
| E222 | winter wheat | Euforia | root | greenhouse | *Trichoderma viride* | MZ447575 |  |  |  |  |  |  |
| E223 | winter wheat | Euforia | root | greenhouse | *Trichoderma sp.* | MZ447576 |  |  |  |  |  |  |
| E225 | winter wheat | Euforia | root | greenhouse | *Sarocladium sp.* | MZ447578 |  |  |  |  |  |  |
| E226 | winter wheat | Ostroga | root | conventional | *Setophoma terrestris* | MW775854 | OK328124 | * | * |  |  |  |
| E227 | winter wheat | Ostroga | root | conventional | *Setophoma terrestris* | MW775855 | OK328125 | * |  |  |  |  |
| E228 | winter wheat | Ostroga | root | conventional | *setophaeria pedicellata* | MW775856 |  |  | * |  |  |  |
| E230 | winter wheat | Ostroga | root | conventional | *Fusarium sp.* | MW775857 |  |  |  |  |  |  |
| E231 | winter wheat | Ostroga | root | conventional | *Setophoma terrestris* | MW775858 | OK328126 | * | * |  |  |  |
| E232 | winter wheat | Ostroga | root | conventional | *Periconia macrospinosa* | MW775859 |  | * |  |  |  |  |
| E233 | winter wheat | Ostroga | root | conventional | *Periconia macrospinosa* | MW775860 |  | * |  |  |  |  |
| E234 | winter wheat | Ostroga | root | conventional | *Periconia macrospinosa* | MW775861 |  | * |  |  |  |  |
| E235 | winter wheat | Ostroga | root | conventional | *Setosphaeria pedicellata* | MW775862 |  |  | * |  |  |  |
| E236 | winter wheat | Ostroga | root | conventional | *Setophaeria pedicellata* | MW775863 |  |  | * |  |  |  |
| E237 | winter wheat | Ostroga | root | conventional | *Microdochium bolleyi* | MW775864 |  |  | * |  |  |  |
| E238 | winter wheat | Ostroga | root | conventional | *Fusarium oxysporum* | MW775865 |  |  |  | * |  |  |
| E239 | winter wheat | Ostroga | root | conventional | *Setophaeria pedicellata* | MW775866 |  |  | * |  |  |  |
| E240 | winter wheat | Ostroga | root | conventional | *Setosphaeria pedicellata* | MW775867 |  |  | * |  |  |  |
| E241 | winter wheat | Ostroga | root | conventional | *Fusarium oxysporum* | MW775868 |  |  |  | * |  |  |
| E242 | winter wheat | Ostroga | root | conventional | *Periconia macrospinosa* | MW775869 |  |  |  |  |  |  |
| E243 | winter wheat | Ostroga | root | conventional | *Fusarium sp.* | MW775870 |  |  |  |  |  |  |
| E244 | winter wheat | Ostroga | root | conventional | *Microdochium bolleyi* | MW775871 |  |  | * |  |  |  |
| E245 | winter wheat | Ostroga | root | conventional | *Periconia macrospinosa* | MW775872 |  | * |  |  |  |  |
| E246 | winter wheat | Ostroga | root | conventional | *Fusarium sp.* | MW775873 |  |  |  |  |  |  |
| E247 | winter wheat | Ostroga | root | conventional | *Fusarium oxysporum* | MW775874 |  |  |  | * |  |  |
| E248 | winter wheat | Ostroga | root | conventional | *Periconia macrospinosa* | MW775875 |  |  |  |  |  |  |
| E249 | winter wheat | Ostroga | root | conventional | *Fusarium redolens* | MW775876 |  |  |  | * |  |  |
| E250 | winter wheat | Ostroga | root | conventional | *Periconia macrospinosa* | MW775877 |  | * |  |  |  |  |
| E251 | winter wheat | Ostroga | root | conventional | *Fusarium oxysporum* | MW775878 |  |  |  | * |  |  |
| E252 | winter wheat | Ostroga | root | conventional | *Setophoma terrestris* | MW775879 |  | OK328127 |  |  |  |  |
| E253 | winter wheat | Ostroga | root | conventional | *Fusarium redolens* | MW775880 |  |  |  | * |  |  |
| E254 | winter wheat | Ostroga | root | conventional | *Fusarium oxysporum* | MW775881 |  |  |  | * |  |  |
| E255 | winter wheat | Ostroga | stem | conventional | *Microdochium bolleyi* | MW775882 |  |  | * |  |  |  |
| E256 | winter wheat | Ostroga | stem | conventional | *Cladosporium sp.* | MW775883 |  |  |  |  |  |  |
| E257 | winter wheat | Ostroga | leaf | conventional | *Alternaria sp.* | MW775884 |  |  |  |  |  |  |
| E258 | winter wheat | Ostroga | leaf | conventional | *Fusarium oxysporum* | MW775885 |  |  |  | * |  |  |
| E259 | winter wheat | Arkadia | root | conventional | *Periconia macrospinosa* | MW775886 |  | * |  |  |  |  |
| E261 | winter wheat | Arkadia | root | conventional | *Trichoderma sp.* | MW775888 |  |  |  |  |  |  |
| E262 | winter wheat | Arkadia | root | conventional | *Trichoderma sp.* | MW775889 |  |  |  |  |  |  |
| E263 | winter wheat | Arkadia | root | conventional | *Periconia macrospinosa* | MW775890 |  | * |  |  |  |  |
| E267 | winter wheat | Arkadia | root | conventional | *Periconia macrospinosa* | MW775891 |  | * |  |  |  |  |
| E268 | winter wheat | Arkadia | root | conventional | *Penicilium sp.* | MW775892 |  |  |  |  |  |  |
| E269 | winter wheat | Arkadia | root | conventional | *Microdochium bolleyi* | MW775893 |  |  | * |  |  |  |
| E270 | winter wheat | Arkadia | root | conventional | *Microdochium bolleyi* | MW775894 |  |  | * |  |  |  |
| E271 | winter wheat | Arkadia | root | conventional | *Penicilium sp.* | MW775895 |  |  |  |  |  |  |
| E272 | winter wheat | Arkadia | root | conventional | *Microdochium bolleyi* | MW775896 |  |  |  |  |  |  |
| E273 | winter wheat | Arkadia | root | conventional | *Periconia macrospinosa* | MW775897 |  | OK328128 |  |  |  |  |
| E274 | winter wheat | Arkadia | root | conventional | *Microdochium bolleyi* | MW775898 |  |  |  |  |  |  |
| E275 | winter wheat | Arkadia | root | conventional | *Periconia macrospinosa* | MW775899 |  | * |  |  |  |  |
| E276 | winter wheat | Arkadia | root | conventional | *Periconia macrospinosa* | MW775900 |  |  |  |  |  |  |
| E277 | winter wheat | Arkadia | root | conventional | *Fusarium poae* | MW775901 |  |  |  |  |  |  |
| E278 | winter wheat | Arkadia | root | conventional | *Periconia macrospinosa* | MW775902 |  | OK328129 |  |  |  |  |
| E279 | winter wheat | Arkadia | root | conventional | *Fusarium sp.* | MW775903 |  |  |  |  |  |  |
| E280 | winter wheat | Arkadia | stem | conventional | *Stemphylium vesicarium* | MW775904 |  | OK328130 |  |  |  |  |
| E281 | winter wheat | Arkadia | stem | conventional | *Stemphylium vesicarium* | MW775905 |  | OK328131 |  |  |  |  |
| E282 | winter wheat | Arkadia | stem | conventional | *Alternaria sp.* | MW775906 |  |  |  |  |  |  |
| E283 | winter wheat | Arkadia | stem | conventional | *Stemphylium vesicarium* | MW775907 |  | * |  |  |  |  |
| E284 | winter wheat | Arkadia | stem | conventional | *Stemphylium vesicarium* | MW775908 |  | * |  |  |  |  |
| E285 | winter wheat | Arkadia | stem | conventional | *Stemphylium vesicarium* | MW775909 |  | * | * |  |  |  |
| E286 | winter wheat | Arkadia | leaf | conventional | *Alternaria sp.* | MW775910 |  |  |  |  |  |  |
| E287 | winter wheat | Arkadia | leaf | conventional | *Alternaria sp.* | MW775911 |  |  |  |  |  |  |
| E288 | winter wheat | Arkadia | leaf | conventional | *Alternaria sp.* | MW775912 |  |  |  |  |  |  |
| E289 | winter wheat | Legenda | root | conventional | *Microdochium bolleyi* | MW775913 |  |  |  |  |  |  |
| E290 | winter wheat | Legenda | root | conventional | *Fusarium poae* | MW775914 |  |  |  |  |  |  |
| E291 | winter wheat | Legenda | root | conventional | *Setophoma terrestris* | MW775915 |  |  | * |  |  |  |
| E292 | winter wheat | Legenda | root | conventional | *Fusarium oxysporum* | MW775916 |  |  |  |  |  |  |
| E293 | winter wheat | Legenda | root | conventional | *Setophoma terrestris* | MW775917 |  | OK328132 |  |  |  |  |
| E294 | winter wheat | Legenda | root | conventional | *Setophoma terrestris* | MW775918 |  | * |  |  |  |  |
| E295 | winter wheat | Legenda | root | conventional | *Microdochium bolleyi* | MW775919 |  |  |  |  |  |  |
| E296 | winter wheat | Legenda | root | conventional | *Fusarium redolens* | MW775920 |  |  |  | * |  |  |
| E297 | winter wheat | Legenda | root | conventional | *Setophoma sp.* | MW775921 |  |  |  |  |  |  |
| E298 | winter wheat | Legenda | root | conventional | *Periconia macrospinosa* | MW888903 |  | * |  |  |  |  |
| E299 | winter wheat | Legenda | root | conventional | *Fusarium poae* | MW775922 |  |  |  | * |  |  |
| E301 | winter wheat | Legenda | root | conventional | *Periconia macrospinosa* | MW775924 |  | * |  |  |  |  |
| E302 | winter wheat | Legenda | root | conventional | *Setophoma terrestris* | MW775925 |  | OK328133 |  |  |  |  |
| E303 | winter wheat | Legenda | leaf | conventional | *Alternaria sp.* | MW775926 |  |  |  |  |  |  |
| E304 | winter wheat | Legenda | leaf | conventional | *Alternaria sp.* | MW775927 |  |  |  |  |  |  |
| E305 | winter wheat | Legenda | leaf | conventional | *Alternaria sp.* | MW775928 |  |  |  |  |  |  |
| E306 | winter wheat | Legenda | leaf | conventional | *Alternaria sp.* | MW775929 |  |  |  |  |  |  |
| E307 | winter wheat | Legenda | leaf | conventional | *Alternaria sp.* | MW775930 |  |  |  |  |  |  |
| E308 | winter wheat | Legenda | leaf | conventional | *Alternaria sp.* | MW775931 |  |  |  |  |  |  |
| E309 | winter wheat | Legenda | leaf | conventional | *Alternaria sp.* | MW775932 |  |  |  |  |  |  |
| E310 | winter wheat | Legenda | leaf | conventional | *Alternaria sp.* | MW775933 |  |  |  |  |  |  |
| E311 | winter wheat | Legenda | leaf | conventional | *Periconia macrospinosa* | MW775934 |  | * |  |  |  |  |
| E312 | winter wheat | Legenda | leaf | conventional | *Penicilium amphipolaria* | MW775935 |  |  | * |  |  |  |
| E313 | winter wheat | Legenda | stem | conventional | *Fusarium sp.* | MW775936 |  |  |  | * |  |  |
| E314 | winter wheat | Legenda | stem | conventional | *Fusarium sp.* | MW775937 |  |  |  | * |  |  |
| E315 | winter wheat | Legenda | stem | conventional | *Sarocladium strictum* | MW775938 |  |  |  |  |  |  |
| E316 | winter wheat | Legenda | stem | conventional | *Rhizoctonia solani* | MW775939 |  |  |  |  |  |  |
| E317 | winter wheat | Legenda | stem | conventional | *Fusarium sp.* | MW775940 |  |  |  | * |  |  |
| E318 | winter wheat | Legenda | stem | conventional | *Fusarium sp.* | MW775941 |  |  |  | * |  |  |
| E319 | winter wheat | Legenda | stem | conventional | *Rhizoctonia solani* | MW775942 |  |  |  |  |  |  |
| E320 | winter wheat | Euforia | root | conventional | *Setosphaeria pedicellata* | MW775943 |  |  | * |  |  |  |
| E321 | winter wheat | Euforia | root | conventional | *Microdochium bolleyi* | MW775944 |  |  |  |  |  |  |
| E322 | winter wheat | Euforia | root | conventional | *Fusarium redolens* | MW775945 |  |  |  | * |  |  |
| E323 | winter wheat | Euforia | root | conventional | *Setophoma terrestris* | MW775946 |  | * | * |  |  |  |
| E324 | winter wheat | Euforia | root | conventional | *Fusarium oxysporum* | MW775947 |  |  |  | * |  |  |
| E325 | winter wheat | Euforia | root | conventional | *Setophoma terrestris* | MW775948 |  | OK328134 | * |  |  |  |
| E326 | winter wheat | Euforia | root | conventional | *Fusarium sp.* | MW775949 |  |  |  |  |  |  |
| E327 | winter wheat | Euforia | root | conventional | *Setosphaeria pedicellata* | MW775950 |  |  | * |  |  |  |
| E328 | winter wheat | Euforia | root | conventional | *Fusarium redolens* | MW775951 |  |  |  | * |  |  |
| E329 | winter wheat | Euforia | root | conventional | *Fusarium oxysporum* | MW775952 |  |  |  | * |  |  |
| E330 | winter wheat | Euforia | root | conventional | *Setophoma terrestris* | MW775953 |  | OK328135 |  |  |  |  |
| E331 | winter wheat | Euforia | root | conventional | *Cladosporium sp.* | MW775954 |  |  | * |  |  |  |
| E332 | winter wheat | Euforia | root | conventional | *Fusarium poae* | MW775955 |  |  |  | * |  |  |
| E333 | winter wheat | Euforia | root | conventional | *Fusarium poae* | MW775956 |  |  |  | * |  |  |
| E334 | winter wheat | Euforia | root | conventional | *Microdochium sp.* | MW775957 |  |  | * |  |  |  |
| E335 | winter wheat | Euforia | root | conventional | *Fusarium sp.* | MW775958 |  |  |  |  |  |  |
| E336 | winter wheat | Euforia | root | conventional | *Setosphaeria pedicellata* | MW775959 |  |  | * | * |  |  |
| E337 | winter wheat | Euforia | root | conventional | *Setophoma terrestris* | MW775960 |  |  | * | * |  |  |
| E338 | winter wheat | Euforia | root | conventional | *Fusarium avenaceum* | MW775961 |  |  |  | * |  |  |
| E339 | winter wheat | Euforia | leaf | conventional | *Alternaria sp.* | MW775962 |  |  |  |  |  |  |
| E340 | winter wheat | Euforia | leaf | conventional | *Alternaria sp.* | MW775963 |  |  |  |  |  |  |
| E341 | winter wheat | Euforia | leaf | conventional | *Alternaria sp.* | MW775964 |  |  |  |  |  |  |
| E342 | winter wheat | Euforia | leaf | conventional | *Alternaria sp.* | MW775965 |  |  |  |  |  |  |
| E343 | winter wheat | Euforia | leaf | conventional | *Alternaria sp.* | MW775966 |  |  |  |  |  |  |
| E344 | winter wheat | Euforia | leaf | conventional | *Alternaria sp.* | MW775967 |  |  |  | * |  |  |
| E346 | winter wheat | Euforia | stem | conventional | *Setosphaeria pedicellata* | MW775969 |  |  | * | * |  |  |
| E347 | winter wheat | Euforia | seed | conventional | *Cladosporium sp.* | MW775970 |  |  |  |  |  |  |
| E348 | winter wheat | Euforia | seed | conventional | *Sarocladium sp.* | MW775971 |  |  |  |  |  |  |
| E349 | winter wheat | Bamberka | root | conventional | *Setophoma terrestris* | MW775972 |  | OK328136 |  |  |  |  |
| E350 | winter wheat | Bamberka | root | conventional | *Microdochium bolleyi* | MW775973 |  |  |  |  |  |  |
| E351 | winter wheat | Bamberka | root | conventional | *Fusarium sp.* | MW775974 |  |  |  |  |  |  |
| E352 | winter wheat | Bamberka | root | conventional | *Fusarium redolens* | MW775975 |  |  |  | * |  |  |
| E353 | winter wheat | Bamberka | root | conventional | *Fusarium oxysporum* | MW775976 |  |  |  | * |  |  |
| E354 | winter wheat | Bamberka | root | conventional | *Setophoma terrestris* | MW775977 |  | OK328137 | * |  |  |  |
| E355 | winter wheat | Bamberka | root | conventional | *Fusarium redolens* | MW775978 |  |  |  | * |  |  |
| E356 | winter wheat | Bamberka | root | conventional | *Penicillium sp.* | MW775979 |  |  |  |  |  |  |
| E358 | winter wheat | Bamberka | root | conventional | *Phoma sp.* | MW775981 |  |  |  |  |  |  |
| E359 | winter wheat | Bamberka | root | conventional | *Michrodochium bolleyi* | MW775982 |  |  |  |  |  |  |
| E360 | winter wheat | Bamberka | root | conventional | *Fusarium sp.* | MW775983 |  |  |  |  |  |  |
| E361 | winter wheat | Bamberka | root | conventional | *Fusarium sp.* | MW775984 |  |  |  | * |  |  |
| E362 | winter wheat | Bamberka | root | conventional | *Penicillium sp.* | MW775985 |  |  | * |  |  |  |
| E363 | winter wheat | Bamberka | root | conventional | *Fusarium oxysporum* | MW775986 |  |  |  | * |  |  |
| E364 | winter wheat | Bamberka | root | conventional | *Periconia macrospinosa* | MW775987 |  | * |  |  |  |  |
| E365 | winter wheat | Bamberka | root | conventional | *Backusella sp.* | MW775988 |  |  | * |  |  |  |
| E366 | winter wheat | Bamberka | leaf | conventional | *Phoma sp.* | MW775989 |  |  |  |  |  |  |
| E367 | winter wheat | Bamberka | leaf | conventional | *Setosphaeria pedicellata* | MW775990 |  |  | * |  |  |  |
| E368 | winter wheat | Legenda | root | conventional | *Epicoccum sp.* | MW775991 |  |  |  |  |  |  |
| E369 | winter wheat | Legenda | root | conventional | *Setosphaeria pedicellata* | MW775992 |  |  | * |  |  |  |
| E370 | winter wheat | Legenda | root | conventional | *Penicillium sp.* | MW775993 |  |  | * |  |  |  |
| E371 | winter wheat | Legenda | root | conventional | *Microdochium bolleyi* | MW775994 |  | * | * |  |  |  |
| E372 | winter wheat | Legenda | root | conventional | *Microdochium bolleyi* | MW775995 |  | * |  |  |  |  |
| E373 | winter wheat | Legenda | root | conventional | *Setophoma terrestris* | MW775996 |  | OK328139 |  |  |  |  |
| E374 | winter wheat | Legenda | root | conventional | *Setosphaeria pedicella* | MW775997 |  |  | * |  |  |  |
| E375 | winter wheat | Legenda | root | conventional | *Fusarium sp.* | MW775998 |  |  |  |  |  |  |
| E376 | winter wheat | Legenda | root | conventional | *Fusarium redolens* | MW775999 |  |  |  | * |  |  |
| E377 | winter wheat | Legenda | root | conventional | *Fusarium redolens* | MW776000 |  |  |  | * |  |  |
| E378 | winter wheat | Legenda | root | conventional | *Fusarium avenaceum* | MW776001 |  |  |  | * |  |  |
| E379 | winter wheat | Legenda | root | conventional | *Epicoccum sp.* | MW776002 |  |  |  |  |  |  |
| E380 | winter wheat | Legenda | root | conventional | *Fusarium sp.* | MW776003 |  |  |  |  |  |  |
| E381 | winter wheat | Legenda | root | conventional | *Fusarium redolens* | MW776004 |  |  |  | * |  |  |
| E382 | winter wheat | Legenda | root | conventional | *Fusarium sp.* | MW776005 |  |  |  | * |  |  |
| E383 | winter wheat | Legenda | leaf | conventional | *Alternaria sp.* | MW776006 |  |  |  |  |  |  |
| E384 | winter wheat | Legenda | leaf | conventional | *Alternaria sp.* | MW776007 |  |  |  |  |  |  |
| E385 | winter wheat | Legenda | leaf | conventional | *Phoma sp.* | MW776008 |  |  |  |  |  |  |
| E386 | winter wheat | Legenda | leaf | conventional | *Phoma sp.* | MW776009 |  |  | * |  |  |  |
| E387 | winter wheat | Legenda | leaf | conventional | *Phoma sp.* | MW776010 |  |  | * |  |  |  |
| E388 | winter wheat | Legenda | stem | conventional | *Cadophora sp.* | MW776011 |  | * |  |  |  |  |
| E389 | winter wheat | Arkadia | root | conventional | *Microdochium bolleyi* | MW776012 |  | * |  |  |  |  |
| E390 | winter wheat | Arkadia | root | conventional | *Phoma eupyrena* | MW776013 |  | * |  |  |  |  |
| E391 | winter wheat | Arkadia | root | conventional | *Setophoma terrestris* | MW776014 |  | OK328140 | * |  |  |  |
| E392 | winter wheat | Arkadia | root | conventional | *Setophoma terrestris* | MW776015 |  | OK328141 | * |  |  |  |
| E394 | winter wheat | Arkadia | root | conventional | *Fusarium sp.* | MW776016 |  |  |  |  |  |  |
| E395 | winter wheat | Arkadia | root | conventional | *Microdochium bolleyi* | MW776017 |  | * | * |  |  |  |
| E396 | winter wheat | Arkadia | root | conventional | *Setosphaeria pedicellata* | MW776018 |  |  | * |  |  |  |
| E397 | winter wheat | Arkadia | root | conventional | *Fusarium sp.* | MW776019 |  |  |  |  |  |  |
| E399 | winter wheat | Arkadia | root | conventional | *Fusarium avenaceum* | MW776021 |  |  |  | * |  |  |
| E400 | winter wheat | Arkadia | root | conventional | *Fusarium avenaceum* | MW776022 |  |  |  |  |  |  |
| E401 | winter wheat | Arkadia | leaf | conventional | *Penicillium sp.* | MW776023 |  |  | * |  |  |  |
| E402 | winter wheat | Arkadia | leaf | conventional | *Penicillium sp.* | MW776024 |  |  | * |  |  |  |
| E403 | winter wheat | Ostroga | root | conventional | *Setosphaeria pedicellata* | MW776025 |  |  | * |  |  |  |
| E404 | winter wheat | Ostroga | root | conventional | *Bipolaris sorokiniana* | MW776026 |  | * |  |  |  |  |
| E405 | winter wheat | Ostroga | root | conventional | *Fusarium oxysporum* | MW776027 |  |  |  | * |  |  |
| E406 | winter wheat | Ostroga | root | conventional | *Microdochium bolleyi* | MW776028 |  | * | * |  |  |  |
| E407 | winter wheat | Ostroga | root | conventional | *Setophoma terrestris* | MW776029 |  |  |  |  |  |  |
| E408 | winter wheat | Ostroga | root | conventional | *Setophoma terrestris* | MW776030 |  |  |  |  |  |  |
| E409 | winter wheat | Ostroga | root | conventional | *Microdochium bolleyi* | MW776031 |  | * | * |  |  |  |
| E410 | winter wheat | Ostroga | root | conventional | *Microdochium bolleyi* | MW776032 |  | * | * |  |  |  |
| E411 | winter wheat | Ostroga | root | conventional | *Fusarium oxysporum* | MW776033 |  |  |  | * |  |  |
| E412 | winter wheat | Ostroga | root | conventional | *Penicillium sp.* | MW776034 |  |  | * |  |  |  |
| E413 | winter wheat | Ostroga | root | conventional | *Penicillium sp.* | MW776035 |  |  | * |  |  |  |
| E414 | winter wheat | Ostroga | root | conventional | *Setosphaeria pedicellata* | MW776036 |  |  | * |  |  |  |
| E415 | winter wheat | Ostroga | root | conventional | *Bipolaris sorokiniana* | MW776037 |  | * |  |  |  |  |
| E416 | winter wheat | Ostroga | root | conventional | *Setophoma terrestris* | MW776038 |  | OK328142 |  |  |  |  |
| E417 | winter wheat | Ostroga | stem | conventional | *Michrodochium bolleyi* | MW776039 |  |  |  |  |  |  |
| E418 | winter wheat | Bamberka | root | conventional | *Setosphaeria pedicellata* | MW776040 |  |  | * |  |  |  |
| E419 | winter wheat | Bamberka | root | conventional | *Setosphaeria pedicellata* | MW776041 |  |  | * |  |  |  |
| E420 | winter wheat | Bamberka | root | conventional | *Setophoma terrestris* | MW776042 |  | OK328143 | * | * |  |  |
| E421 | winter wheat | Bamberka | root | conventional | *Setophoma terrestris* | MW776043 |  | OK328144 |  |  |  |  |
| E422 | winter wheat | Bamberka | root | conventional | *Setophoma terrestris* | MW776044 |  | OK328145 |  |  |  |  |
| E423 | winter wheat | Bamberka | root | conventional | *Microdochium bolleyi* | MW776045 |  | * | * |  |  |  |
| E424 | winter wheat | Bamberka | root | conventional | *Setophoma terrestris* | MW776046 |  | OK328146 |  |  |  |  |
| E425 | winter wheat | Bamberka | root | conventional | *Microdochium bolleyi* | MW776047 |  | * |  |  |  |  |
| E426 | winter wheat | Bamberka | root | conventional | *Fusarium oxysporum* | MW776048 |  |  |  | * |  |  |
| E427 | winter wheat | Bamberka | root | conventional | *Fusarium oxysporum* | MW776049 |  |  |  | * |  |  |
| E428 | winter wheat | Bamberka | root | conventional | *Fusarium oxysporum* | MW776050 |  |  |  |  |  |  |
| E429 | winter wheat | Bamberka | root | conventional | *Microdochium bolley* | MW776051 |  | * | * |  |  |  |
| E430 | winter wheat | Bamberka | root | conventional | *Setosphaeria pedicellata* | MW776052 |  |  | * | * |  |  |
| E431 | winter wheat | Bamberka | stem | conventional | *Penicillium chrysogenum* | MW776053 |  |  | * |  |  |  |
| E432 | winter wheat | Euforia | root | conventional | *Setophoma terrestris* | MW776054 |  | OK328147 | * |  |  |  |
| E433 | winter wheat | Euforia | root | conventional | *Phoma sp.* | MW776055 |  |  | * |  |  |  |
| E434 | winter wheat | Euforia | root | conventional | *Fusarium avenaceum* | MW776056 |  |  |  | * |  |  |
| E435 | winter wheat | Euforia | root | conventional | *Fusarium avenaceum* | MW776057 |  |  |  | * |  |  |
| E436 | winter wheat | Euforia | root | conventional | *Setophoma terrestris* | MW776058 |  | OK328148 |  |  |  |  |
| E437 | winter wheat | Euforia | root | conventional | *Phoma sp.* | MW776059 |  |  |  |  |  |  |
| E438 | winter wheat | Euforia | root | conventional | *Fusarium oxysporum* | MW776060 |  |  |  | * |  |  |
| E439 | winter wheat | Euforia | root | conventional | *Periconia macrospinosa* | MW776061 |  |  |  | * |  |  |
| E440 | winter wheat | Euforia | root | conventional | *Fusarium oxysporum* | MW776062 |  |  |  | * |  |  |
| E441 | winter wheat | Euforia | root | conventional | *Fusarium sp.* | MW776063 |  |  |  |  |  |  |
| E442 | winter wheat | Euforia | root | conventional | *Periconia macrospinosa* | MW776064 |  | * |  |  |  |  |
| E443 | winter wheat | Euforia | seed | conventional | *Alternaria sp.* | MW776065 |  |  |  | * |  |  |
| E444 | winter wheat | Euforia | seed | conventional | *Alternaria sp.* | MW776066 |  |  |  |  |  |  |
| E446 | spring wheat | Rusałka | root | conventional | *Fusarium oxysporum* | MW776067 |  |  |  | * |  |  |
| E447 | spring wheat | Rusałka | root | conventional | *Penicillium sp.* | MW776068 |  |  |  |  |  |  |
| E448 | spring wheat | Rusałka | root | conventional | *Periconia macrospinosa* | MW776069 |  | * |  |  |  |  |
| E449 | spring wheat | Rusałka | root | conventional | *Periconia macrospinosa* | MW776070 |  |  |  |  |  |  |
| E452 | spring wheat | Rusałka | root | conventional | *Setosphaeria pedicellata* | MW776072 |  |  | * |  |  |  |
| E453 | spring wheat | Rusałka | root | conventional | *Fusarium oxysporum* | MW776073 |  |  |  | * |  |  |
| E454 | spring wheat | Rusałka | stem | conventional | *Periconia macrospinosa* | MW776074 |  |  |  |  |  |  |
| E455 | spring wheat | Rusałka | stem | conventional | *Alternaria sp.* | MW776075 |  |  |  | * |  |  |
| E456 | spring wheat | Rusałka | stem | conventional | *Alternaria sp.* | MW776076 |  |  |  |  |  |  |
| E457 | spring wheat | Rusałka | stem | conventional | *Alternaria alternata* | MW776077 |  |  |  | * |  |  |
| E458 | spring wheat | Rusałka | stem | conventional | *Alternaria infectoria* | MW776078 |  |  | * | * |  |  |
| E459 | spring wheat | Rusałka | seed | conventional | *Penicillium sp.* | MW776079 |  |  | * |  |  |  |
| E460 | spring wheat | Rusałka | seed | conventional | *Didymella pomorum* | MW776080 |  |  | * |  |  |  |
| E461 | spring wheat | Rusałka | seed | conventional | *Alternaria infactoria* | MW776081 |  |  |  | * |  |  |
| E462 | spring wheat | Rospuda | root | conventional | *Setophoma terrestris* | MW776082 |  | OK328149 | * |  |  |  |
| E463 | spring wheat | Rospuda | root | conventional | *Phoma pomorum* | MW776083 |  |  | * |  |  |  |
| E464 | spring wheat | Rospuda | root | conventional | *Arthrinium sp.* | MW776084 |  |  | * |  |  |  |
| E465 | spring wheat | Rospuda | root | conventional | *Periconia macrospinosa* | MW776085 |  | * | * |  |  |  |
| E466 | spring wheat | Rospuda | seed | conventional | *Cladosporium sp.* | MW776086 |  |  |  |  |  |  |
| E467 | spring wheat | Rospuda | seed | conventional | *Penicillium sp.* | MW776087 |  |  | * |  |  |  |
| E468 | spring wheat | Rospuda | seed | conventional | *Cladosporium sp.* | MW776088 |  |  | * |  |  |  |
| E469 | spring wheat | Rospuda | seed | conventional | *Cladosporium sp.* | MW776089 |  |  | * |  |  |  |
| E470 | spring wheat | Kandela | root | conventional | *Phoma pomorum* | MW776090 |  |  | * |  |  |  |
| E471 | spring wheat | Kandela | root | conventional | *Setophoma terrestris* | MW776091 |  | OK328150 | * |  |  |  |
| E472 | spring wheat | Kandela | root | conventional | *Setosphaeria pedicellat* | MW776092 |  |  | * |  |  |  |
| E473 | spring wheat | Kandela | root | conventional | *Fusarium oxysporum* | MW776093 |  |  |  | * |  |  |
| E474 | spring wheat | Kandela | root | conventional | *Phoma sp.* | MW776094 |  |  |  |  |  |  |
| E475 | spring wheat | Kandela | root | conventional | *Penicillium sp.* | MW776095 |  |  | * |  |  |  |
| E476 | spring wheat | Kandela | root | conventional | *Penicillium sp.* | MW776096 |  |  | * | * |  |  |
| E477 | spring wheat | Kandela | root | conventional | *Penicillium sp.* | MW776097 |  |  | * |  |  |  |
| E478 | spring wheat | Kandela | root | conventional | *Setosphaeria pedicellata* | MW776098 |  |  | * |  |  |  |
| E479 | spring wheat | Kandela | root | conventional | *Phoma pomorum* | MW776099 |  |  | * |  |  |  |
| E480 | spring wheat | Kandela | root | conventional | *Setophoma terrestris* | MW776100 |  | OK328151 | * |  |  |  |
| E481 | spring wheat | Kandela | root | conventional | *Penicilium sp.* | MW776101 |  |  |  |  |  |  |
| E482 | spring wheat | Kandela | stem | conventional | *Alternaria sp.* | MW776102 |  |  |  |  |  |  |
| E483 | spring wheat | Kandela | stem | conventional | *Fusarium sp.* | MW776103 |  |  |  |  |  |  |
| E484 | spring wheat | Kandela | stem | conventional | *Penicillium sp.* | MW776104 |  |  | * |  |  |  |
| E485 | spring wheat | Kandela | leaf | conventional | *Alternaria sp.* | MW776105 |  |  | * |  |  |  |
| E486 | spring wheat | Kandela | seed | conventional | *Penicillium sp.* | MW776106 |  |  |  |  |  |  |
| E487 | winter wheat | Ostroga | root | conventional | *Setophoma terrestris* | MW776107 |  | * |  |  |  |  |
| E488 | winter wheat | Ostroga | root | conventional | *Setophoma terrestris* | MW776108 |  |  | * |  |  |  |
| E489 | winter wheat | Ostroga | root | conventional | *Fusarium oxysporum* | MW776109 |  |  |  | * |  |  |
| E490 | winter wheat | Ostroga | leaf | conventional | *Fusarium oxysporum* | MW776110 |  |  |  |  |  |  |
| E491 | winter wheat | Ostroga | leaf | conventional | *Alternaria sp.* | MW776111 |  |  |  |  |  |  |
| E492 | winter wheat | Ostroga | leaf | conventional | *Fusarium oxysporum* | MW776112 |  |  |  | * |  |  |
| E493 | winter wheat | Arkadia | leaf | conventional | *Anthracocystis sp.* | MW776113 |  |  |  |  |  |  |
| E494 | winter wheat | Arkadia | leaf | conventional | *Moesziomyces sp.* |  |  |  | * |  |  |  |
| E495 | winter wheat | Arkadia | seed | conventional | *Anthracocystis flocculosa* | MW776114 |  |  |  |  |  |  |
| E496 | winter wheat | Arkadia | seed | conventional | *Anthracocystis flocculosa* | MW776115 |  |  |  |  |  |  |
| E497 | winter wheat | Legenda | root | conventional | *Microdochium bolleyi* | MW776116 |  | * | * |  |  |  |
| E499 | winter wheat | Legenda | leaf | conventional | *Fusarium sp.* | MW776118 |  |  |  | * |  |  |
| E500 | winter wheat | Legenda | leaf | conventional | *Sarocladium strictum* | MW776119 |  |  |  |  |  |  |
| E501 | winter wheat | Legenda | stem | conventional | *Fusarium sp.* | MW776120 |  |  |  | * |  |  |
| E502 | winter wheat | Legenda | stem | conventional | *Sarocladium strictum* | MW776121 |  |  |  | * |  |  |
| E503 | winter wheat | Euforia | root | conventional | *Periconia macrospinosa* | MW776122 |  | * |  |  |  |  |
| E504 | winter wheat | Euforia | root | conventional | *Periconia macrospinosa* | MW776123 |  |  |  |  |  |  |
| E505 | winter wheat | Euforia | root | conventional | *Setophoma terrestris* | MW776124 |  | * | * |  |  |  |
| E506 | winter wheat | Euforia | root | conventional | *Setophoma terrestris* | MW776125 |  | * | * |  |  |  |
| E507 | winter wheat | Bamberka | root | conventional | *Setophoma terrestris* | MW776126 |  | * |  |  |  |  |
| E508 | winter wheat | Bamberka | root | conventional | *Fusarium oxysporum* | MW776127 |  |  |  |  |  |  |
| E509 | winter wheat | Bamberka | root | conventional | *Setophoma terrestris* | MW776128 |  | * | * |  |  |  |
| E511 | winter wheat | Bamberka | root | conventional | *Setophoma terrestris* | MW776129 |  | * |  |  |  |  |
| E512 | winter wheat | Bamberka | root | conventional | *Setophoma terrestris* | MW776130 |  | * |  |  |  |  |
| E513 | winter wheat | Bamberka | root | conventional | *Penicillium sp.* | MW776131 |  |  |  |  |  |  |
| E514 | winter wheat | Bamberka | root | conventional | *Neonectria sp.* | MW776132 |  | * | * |  |  |  |
| E515 | winter wheat | Bamberka | root | conventional | *Neonectria sp.* | MW776133 |  | * | * |  |  |  |
| E516 | winter wheat | Bamberka | root | conventional | *Fusarium oxysporum* | MW776134 |  |  |  | * |  |  |
| E517 | winter wheat | Bamberka | root | conventional | *Penicillium sp.* | MW776135 |  |  |  |  |  |  |
| E518 | winter wheat | Bamberka | root | conventional | *Fusarium oxysporum* | MW776136 |  |  |  | * |  |  |
| E519 | winter wheat | Bamberka | leaf | conventional | *Setosphaeria pedicellata* | MW776137 |  |  | * |  |  |  |
| E520 | winter wheat | Bamberka | leaf | conventional | *Setosphaeria pedicellata* | MW776138 |  |  | * |  |  |  |
| E521 | winter wheat | Bamberka | leaf | conventional | *Alternaria sp.* | MW776139 |  |  |  |  |  |  |
| E522 | winter wheat | Bamberka | stem | conventional | *Fusarium sp.* | MW776140 |  |  |  | * |  |  |
| E523 | winter wheat | Legenda | root | no till | *Microdochium bolleyi* | MW776141 |  |  |  |  |  |  |
| E524 | winter wheat | Legenda | root | no till | *Microdochium bolleyi* | MW776142 |  | * | * |  |  |  |
| E525 | winter wheat | Legenda | root | no till | *Fusarium avenaceum* | MW776143 |  |  |  | * |  |  |
| E526 | winter wheat | Ostroga | root | no till | *Setosphaeria pedicellata* | MW776144 |  |  | * |  |  |  |
| E527 | winter wheat | Ostroga | leaf | no till | *Anthracocystis sp.* | MW776145 |  |  |  |  |  |  |
| E528 | winter wheat | Ostroga | leaf | no till | *Cladosporium sp.* | MW776146 |  |  | * |  |  |  |
| E529 | winter wheat | Ostroga | stem | no till | *Anthracocystis sp.* | MW776147 |  |  |  |  |  |  |
| E530 | winter wheat | Ostroga | stem | no till | *Microdochium bolleyi* | MW776148 |  |  |  |  |  |  |
| E531 | winter wheat | Ostroga | seed | no till | *Moesziomyces sp.* | MW776149 |  | * |  |  |  |  |
| E532 | winter wheat | Bamberka | root | no till | *Setophoma terrestris* | MW776150 |  | * | * |  |  |  |
| E533 | winter wheat | Bamberka | root | no till | *Fusarium sp.* | MW776151 |  |  |  | * |  |  |
| E534 | winter wheat | Bamberka | root | no till | *Michrodochium bolleyi* | MW776152 |  |  |  |  |  |  |
| E535 | winter wheat | Bamberka | root | no till | *Periconia macrospinosa* | MW776153 |  |  |  |  |  |  |
| E536 | winter wheat | Bamberka | root | no till | *Periconia macrospinosa* | MW776154 |  | * |  |  |  |  |
| E537 | winter wheat | Bamberka | root | no till | *Setophoma terrestris* | MW776155 |  | * | * |  |  |  |
| E538 | winter wheat | Bamberka | root | no till | *Meyerozyma sp.* | MW776156 |  | * |  |  |  |  |
| E539 | winter wheat | Bamberka | root | no till | *Periconia macrospinosa* | MW776157 |  | * |  |  |  |  |
| E540 | winter wheat | Bamberka | root | no till | *Meyerozyma sp.* | MW776158 |  | * |  |  |  |  |
| E541 | winter wheat | Bamberka | root | no till | *Fusarium oxysporum* | MW888904 |  |  |  | * |  |  |
| E542 | winter wheat | Bamberka | root | no till | *Fusarium sp.* | MW776159 |  |  |  |  |  |  |
| E543 | winter wheat | Bamberka | root | no till | *Setophoma terrestris* | MW776160 |  | OK328152 | * |  |  |  |
| E544 | winter wheat | Bamberka | root | no till | *Colletotrichum sp.* | MW776161 |  |  |  |  |  |  |
| E546 | winter wheat | Bamberka | stem | no till | *Penicillium chrysogenum* | MW776162 |  |  |  | * |  |  |
| E547 | winter wheat | Bamberka | seed | no till | *Alternaria sp.* | MW776163 |  |  |  |  |  |  |
| E548 | winter wheat | Euforia | root | no till | *Periconia macrospinosa* | MW776164 |  | * |  |  |  |  |
| E549 | winter wheat | Euforia | seed | no till | *Alternaria sp.* | MW776165 |  |  |  |  |  |  |
| E550 | spring wheat | Rusałka | root | no till | *Microdochium bolleyi* | MW776166 |  |  | * |  |  |  |
| E551 | spring wheat | Rusałka | root | no till | *Microdochium bolleyi* | MW776167 |  |  | * |  |  |  |
| E552 | spring wheat | Rusałka | root | no till | *Periconia macrospinosa* |  |  | * |  |  |  |  |
| E554 | spring wheat | Rusałka | root | no till | *Penicillium sp.* | MW776169 |  |  | * |  |  |  |
| E555 | spring wheat | Rusałka | root | no till | *Fusarium oxysporum* | MW776170 |  |  |  | * |  |  |
| E556 | spring wheat | Rusałka | leaf | no till | *Cladosporium sp.* | MW776171 |  |  | * |  |  |  |
| E557 | spring wheat | Rusałka | seed | no till | *Anthracocystis sp.* | MW776172 |  |  |  |  |  |  |
| E558 | spring wheat | Rusałka | seed | no till | *Fusarium sp.* | MW776173 |  |  |  |  |  |  |
| E559 | spring wheat | Rusałka | seed | no till | *Fusarium sp.* | MW776174 |  |  |  | * |  |  |
| E560 | spring wheat | Rusałka | seed | no till | *Fusarium sp.* | MW776175 |  |  |  | * |  |  |
| E561 | spring wheat | Rusałka | seed | no till | *Fusarium sp.* | MW776176 |  |  |  | * |  |  |
| E562 | spring wheat | Rospuda | root | no till | *Microdochium bolleyi* | MW888906 |  |  | * |  |  |  |
| E563 | spring wheat | Rospuda | root | no till | *Penicillium chrysogenum* | MW776177 |  |  | * |  |  |  |
| E564 | spring wheat | Rospuda | root | no till | *Fusarium oxysporum* | MW776178 |  |  |  | * |  |  |
| E565 | spring wheat | Rospuda | root | no till | *Setosphaeria pedicellata* | MW776179 |  |  | * |  |  |  |
| E566 | spring wheat | Rospuda | root | no till | *Fusarium oxysporum* | MW776180 |  |  |  | * |  |  |
| E567 | spring wheat | Rospuda | root | no till | *Phoma pomorum* | MW776181 |  |  | * |  |  |  |
| E568 | spring wheat | Rospuda | stem | no till | *Anthracocystis sp.* | MW776182 |  |  |  |  |  |  |
| E569 | spring wheat | Rospuda | stem | no till | *Alternaria sp.* | MW776183 |  |  |  |  |  |  |
| E570 | spring wheat | Rospuda | stem | no till | *Alternaria sp.* | MW776184 |  |  |  |  |  |  |
| E571 | spring wheat | Rospuda | stem | no till | *Aureobasidium pullulans* | MW776185 |  |  | * |  |  |  |
| E572 | spring wheat | Rospuda | stem | no till | *Alternaria alternata* | MW776186 |  |  |  | * |  |  |
| E573 | spring wheat | Rospuda | seed | no till | *Anthracocystis sp.* | MW776187 |  |  |  |  |  |  |
| E574 | spring wheat | Rospuda | seed | no till | *Anthracocystis sp.* | MW776188 |  |  |  |  |  |  |
| E575 | spring wheat | Rospuda | seed | no till | *Penicillium sp.* | MW776189 |  |  | * |  |  |  |
| E576 | spring wheat | Rospuda | seed | no till | *Anthracocystis sp.* | MW776190 |  |  |  |  |  |  |
| E577 | spring wheat | Rospuda | seed | no till | *Anthracocystis sp.* | MW776191 |  |  |  |  |  |  |
| E578 | spring wheat | Kandela | root | no till | *Microdochium bolleyi* | MW776192 |  |  | * |  |  |  |
| E579 | spring wheat | Kandela | root | no till | *Penicillium sp.* | MW776193 |  |  |  |  |  |  |
| E580 | spring wheat | Kandela | root | no till | *Microdochium bolleyi* | MW776194 |  |  | * |  |  |  |
| E581 | spring wheat | Kandela | root | no till | *Setophoma terrestris* | MW776195 |  | OK328153 |  |  |  |  |
| E582 | spring wheat | Kandela | leaf | no till | *Sarocladium sp.* | MW776196 |  |  |  |  |  |  |
| E583 | spring wheat | Kandela | stem | no till | *Sarocladium sp.* | MW776197 |  |  |  |  |  |  |
| E584 | spring wheat | Kandela | stem | no till | *Setophoma terrestris* | MW776198 |  | OK328154 |  |  |  |  |
| E585 | spring wheat | Kandela | stem | no till | *Moesziomyces sp.* | MW776199 |  |  |  |  |  |  |
| E586 | spring wheat | Kandela | stem | no till | *Anthracocystis flocculosa* | MW776200 |  |  |  |  |  |  |
| E587 | spring wheat | Kandela | stem | no till | *Fusarium sp.* | MW776201 |  |  |  | * |  |  |
| E588 | spring wheat | Kandela | seed | no till | *Moesziomyces sp.* | MW776202 |  |  |  |  |  |  |
| E589 | spring wheat | Kandela | seed | no till | *Moesziomyces sp.* | MW776203 |  |  |  |  |  |  |
| E590 | spring wheat | Kandela | seed | no till | *Moesziomyces sp.* | MW776204 |  |  |  |  |  |  |
| E591 | spring wheat | Kandela | seed | no till | *Moesziomyces sp.* | MW776205 |  |  |  |  |  |  |
| E592 | spring wheat | Kandela | seed | no till | *Moesziomyces sp.* | MW776206 |  |  |  |  |  |  |
| E594 | spring wheat | Kandela | seed | no till | *Moesziomyces sp.* | MW776207 |  |  |  |  |  |  |
| E595 | spring wheat | Bombona | root | no till | *Fusarium oxysporum* | MW776208 |  |  |  |  |  |  |
| E596 | spring wheat | Bombona | root | no till | *Fusarium sp.* | MW776209 |  |  |  |  |  |  |
| E597 | spring wheat | Bombona | root | no till | *Fusarium oxysporum* | MW776210 |  |  |  | * |  |  |
| E598 | spring wheat | Bombona | root | no till | *Fusarium sp.* | MW776211 |  |  |  |  |  |  |
| E599 | spring wheat | Bombona | root | no till | *Sarocladium sp.* | MW776212 |  |  |  |  |  |  |
| E600 | spring wheat | Bombona | root | no till | *Penicillium sp.* | MW776213 |  |  |  |  |  |  |
| E601 | spring wheat | Bombona | root | no till | *Fusarium sp.* | MW776214 |  |  |  |  |  |  |
| E602 | spring wheat | Bombona | root | no till | *Waitea circinata* |  |  | * | * |  |  |  |
| E603 | spring wheat | Bombona | root | no till | *Fusarium oxysporum* | MW776215 |  |  |  | * |  |  |
| E604 | spring wheat | Bombona | root | no till | *Fusarium sp.* | MW776216 |  |  |  |  |  |  |
| E605 | spring wheat | Bombona | root | no till | *Fusarium oxysporum* | MW776217 |  |  |  | * |  |  |
| E606 | spring wheat | Bombona | root | no till | *Fusarium sp.* | MW776218 |  |  |  |  |  |  |
| E607 | spring wheat | Bombona | root | no till | *Penicillium sp.* | MW776219 |  |  | * |  |  |  |
| E608 | spring wheat | Bombona | leaf | no till | *Anthracocystis sp.* | MW776220 |  |  |  |  |  |  |
| E609 | spring wheat | Bombona | stem | no till | *Anthracocystis sp.* | MW776221 |  |  |  |  |  |  |
| E610 | spring wheat | Bombona | stem | no till | *Leptobacillium leptobactrum* | MW776222 |  | OK328155 |  |  |  |  |
| E611 | spring wheat | Bombona | stem | no till | *Alternaria sp.* | MW776223 |  |  |  | * |  |  |
| E612 | spring wheat | Bombona | stem | no till | *Verticillium sp.* | MW776224 |  | OK328156 |  |  |  |  |
| E613 | spring wheat | Bombona | stem | no till | *Alternaria sp.* | MW776225 |  |  |  |  |  |  |
| E614 | spring wheat | Bombona | stem | no till | *Alternaria sp.* | MW776226 |  |  |  |  |  |  |
| E615 | spring wheat | Bombona | stem | no till | *Talaromyces sp.* | MW776227 |  |  | * |  |  |  |
| E616 | spring wheat | Bombona | seed | no till | *Didymella sp.* | MW776228 |  |  | * |  |  |  |
| E617 | spring wheat | Bombona | seed | no till | *Penicilium sp.* | MW776229 |  |  | * |  |  |  |
| E618 | spring wheat | Arabella | root | no till | *Fusarium oxysporum* | MW776230 |  |  |  |  |  |  |
| E619 | spring wheat | Arabella | root | no till | *Fusarium sp.* | MW776231 |  |  |  |  |  |  |
| E620 | spring wheat | Arabella | root | no till | *Fusarium oxysporum* | MW776232 |  |  |  | * |  |  |
| E621 | spring wheat | Arabella | root | no till | *Fusarium oxysporum* | MW776233 |  |  |  |  |  |  |
| E624 | spring wheat | Arabella | root | no till | *Fusarium oxysporum* | MW776235 |  |  |  | * |  |  |
| E625 | spring wheat | Arabella | root | no till | *Fusarium solani* | MW776236 |  |  |  | * |  |  |
| E626 | spring wheat | Arabella | leaf | no till | *Penicilium sp.* | MW776237 |  |  |  |  |  |  |
| E628 | spring wheat | Bombona | root | conventional | *Fusarium solani* | MW776238 |  |  |  | * |  |  |
| E629 | spring wheat | Bombona | root | conventional | *Setosphaeria pedicellata* | MW776239 |  |  | * |  |  |  |
| E630 | spring wheat | Bombona | root | conventional | *Penicillium sp.* | MW776240 |  |  |  |  |  |  |
| E632 | spring wheat | Bombona | root | conventional | *Fusarium oxysporum* | MW776241 |  |  |  | * |  |  |
| E633 | spring wheat | Bombona | root | conventional | *Fusarium oxysporum* | MW776242 |  |  |  | * |  |  |
| E634 | spring wheat | Bombona | root | conventional | *Setosphaeria pedicellata* | MW776243 |  |  | * |  |  |  |
| E635 | spring wheat | Bombona | root | conventional | *Fusarium oxysporum* | MW776244 |  |  |  | * |  |  |
| E636 | spring wheat | Bombona | root | conventional | *Setosphaeria pedicellata* | MW776245 |  |  | * |  |  |  |
| E637 | spring wheat | Bombona | leaf | conventional | *Penicillium crustosum* | MW776246 |  |  | * |  |  |  |
| E638 | spring wheat | Bombona | stem | conventional | *Penicilium sp.* | MW776247 |  |  |  |  |  |  |
| E639 | spring wheat | Bombona | seed | conventional | *Alternaria sp.* | MW776248 |  |  |  |  |  |  |
| E640 | spring wheat | Bombona | seed | conventional | *Penicilium sp.* | MW776249 |  |  |  |  |  |  |
| E641 | spring wheat | Rusałka | root | conventional | *Penicilium sp.* | MW776250 |  |  |  |  |  |  |
| E642 | spring wheat | Rusałka | root | conventional | *Fusarium oxysporum* | MW776251 |  |  |  | * |  |  |
| E643 | spring wheat | Rusałka | root | conventional | *Penicilium sp.* | MW776252 |  |  | * |  |  |  |
| E645 | spring wheat | Rusałka | root | conventional | *Fusarium oxysporum* | MW888907 |  |  |  | * |  |  |
| E646 | spring wheat | Rusałka | root | conventional | *Microdochium bolleyi* | MW776254 |  |  | * |  |  |  |
| E648 | spring wheat | Rusałka | root | conventional | *Fusarium sp.* | MW776255 |  |  |  |  |  |  |
| E649 | spring wheat | Rusałka | root | conventional | *Sarocladium strictum* | MW776256 |  |  |  | * |  |  |
| E650 | spring wheat | Rusałka | root | conventional | *Setosphaeria pedicellata* | MW776257 |  |  | * |  |  |  |
| E651 | spring wheat | Rusałka | root | conventional | *Alternaria sp.* | MW776258 |  |  |  |  |  |  |
| E652 | spring wheat | Rusałka | root | conventional | *Achroiostachys betulicola* | MW776259 |  |  | * | * |  |  |
| E653 | spring wheat | Rusałka | leaf | conventional | *Penicillium crustosum* | MW776260 |  |  | * |  |  |  |
| E654 | spring wheat | Rusałka | stem | conventional | *Talaromyces aculeatus* | MW776261 |  |  | * |  |  |  |
| E655 | spring wheat | Rusałka | seed | conventional | *Penicillium crustosum* |  |  |  | * |  |  |  |
| E656 | spring wheat | Rusałka | seed | conventional | *Alternaria sp.* | MW776262 |  |  |  |  |  |  |
| E657 | spring wheat | Rospuda | root | conventional | *Periconia sp.* |  |  |  |  |  |  |  |
| E658 | spring wheat | Rospuda | root | conventional | *Fusarium oxysporum* |  |  |  |  | * |  |  |
| E659 | spring wheat | Rospuda | root | conventional | *Setosphaeria pedicellata* | MW776263 |  |  | * |  |  |  |
| E660 | spring wheat | Rospuda | root | conventional | *Chaetomium sp.* | MW776264 |  |  | * | * |  |  |
| E661 | spring wheat | Rospuda | root | conventional | *Fusarium sp.* | MW776265 |  |  | * |  |  |  |
| E662 | spring wheat | Rospuda | leaf | conventional | *Alternaria conjuncta* | MW776266 |  |  |  | * |  |  |
| E663 | spring wheat | Rospuda | leaf | conventional | *Sarocladium strictum* | MW776267 |  |  |  | * |  |  |
| E664 | spring wheat | Rospuda | stem | conventional | *Alternaria infectoria* | MW776268 |  |  |  | * |  |  |
| E665 | spring wheat | Rospuda | stem | conventional | *Setosphaeria pedicellata* | MW776269 |  |  | * |  |  |  |
| E666 | spring wheat | Rospuda | seed | conventional | *Anthracocystis sp.* | MW776270 |  |  |  |  |  |  |
| E667 | spring wheat | Rospuda | seed | conventional | *Anthracocystis flocculosa* |  |  |  |  | * |  |  |
| E668 | spring wheat | Rospuda | seed | conventional | *Anthracocystis flocculosa* | MW776271 |  |  |  | * |  |  |
| E669 | spring wheat | Rospuda | seed | conventional | *Penicilium sp.* |  |  |  |  |  |  |  |
| E670 | spring wheat | Rospuda | seed | conventional | *Anthracocystis flocculosa* | MW776272 |  |  |  |  |  |  |
| E671 | spring wheat | Kandela | root | conventional | *Fusarium oxysporum* | MW776273 |  |  |  | * |  |  |
| E672 | spring wheat | Kandela | root | conventional | *Fusarium oxysporum* | MW776274 |  |  |  | * |  |  |
| E673 | spring wheat | Kandela | root | conventional | *Bipolaris sorokiniana* | MW776275 |  | OK328157 |  |  |  |  |
| E674 | spring wheat | Kandela | root | conventional | *Fusarium sp.* | MW776276 |  |  |  |  |  |  |
| E675 | spring wheat | Kandela | root | conventional | *Fusarium proliferatum* | MW776277 |  |  |  | * |  |  |
| E676 | spring wheat | Kandela | root | conventional | *Setosphaeria pedicellata* | MW776278 |  |  | * |  |  |  |
| E677 | spring wheat | Kandela | root | conventional | *Periconia macrospinosa* | MW776279 |  | OK328158 |  |  |  |  |
| E678 | spring wheat | Kandela | leaf | conventional | *Alternaria infectoria* | MW776280 |  |  |  | * |  |  |
| E679 | spring wheat | Kandela | leaf | conventional | *Alternaria infectoria* | MW776281 |  |  |  | * |  |  |
| E680 | spring wheat | Kandela | stem | conventional | *Anthracocystis sp.* | MW776282 |  |  |  |  |  |  |
| E681 | spring wheat | Kandela | stem | conventional | *Fusarium sp.* | MW776283 |  |  |  |  |  |  |
| E682 | spring wheat | Arabella | root | conventional | *Fusarium oxysporum* | MW776284 |  |  |  | * |  |  |
| E683 | spring wheat | Arabella | root | conventional | *Penicillium sp.* | MW776285 |  |  | * |  |  |  |
| E684 | spring wheat | Arabella | root | conventional | *Fusarium sp.* | MW776286 |  |  |  |  |  |  |
| E685 | spring wheat | Arabella | root | conventional | *Talaromyces sp.* | MW776287 |  |  |  |  |  |  |
| E686 | spring wheat | Arabella | root | conventional | *Waitea circinata* |  |  | OK328159 |  |  |  |  |
| E688 | spring wheat | Arabella | root | conventional | *Fusarium oxysporum* | MW776288 |  |  |  | * |  |  |
| E689 | spring wheat | Arabella | root | conventional | *Fusarium sp.* | MW776289 |  |  |  |  |  |  |
| E690 | spring wheat | Arabella | root | conventional | *Fusarium oxysporum* | MW776290 |  |  |  | * |  |  |
| E692 | spring wheat | Arabella | stem | conventional | *Fusarium oxysporum* | MW776291 |  |  |  | * |  |  |
| E693 | spring wheat | Arabella | stem | conventional | *Fusarium oxysporum* | MW776292 |  |  |  | * |  |  |
| E694 | spring wheat | Arabella | stem | conventional | *Fusarium oxysporum* | MW776293 |  |  |  | * |  |  |
| E695 | spring wheat | Arabella | stem | conventional | *Fusarium oxysporum* | MW776294 |  |  |  | * |  |  |
| E696 | spring wheat | Arabella | seed | conventional | *Penicilium sp.* | MW776295 |  |  |  |  |  |  |
| E697 | winter wheat | Ostroga | leaf | conventional | *Alternaria sp.* | MW776296 |  |  |  |  |  |  |
| E698 | winter wheat | Ostroga | leaf | conventional | *Fusarium oxysporum* | MW776297 |  |  |  | * |  |  |
| E699 | winter wheat | Arkadia | root | conventional | *Periconia sp.* |  |  |  |  |  |  |  |
| E700 | winter wheat | Legenda | root | conventional | *Setophoma terrestris* |  |  | OK328160 |  |  |  |  |
| E701 | winter wheat | Legenda | root | conventional | *Nigrospora gorlenkoana* | MW776298 |  |  | * |  |  |  |
| E703 | winter wheat | Legenda | root | conventional | *Microdochium bolleyi* | MW776299 |  |  | * |  |  |  |
| E704 | winter wheat | Legenda | root | conventional | *Penicilium sp.* | MW776300 |  |  |  |  |  |  |
| E705 | winter wheat | Legenda | leaf | conventional | *Periconia sp.* | MW776301 |  |  |  |  |  |  |
| E706 | winter wheat | Legenda | leaf | conventional | *Periconia macrospinosa* | MW776302 |  | OK328161 |  |  |  |  |
| E707 | winter wheat | Legenda | leaf | conventional | *Alternaria sp.* | MW776303 |  |  |  |  |  |  |
| E708 | winter wheat | Euforia | root | conventional | *Lecanicillium sp.* | MW776304 |  |  |  |  |  |  |
| E709 | winter wheat | Euforia | leaf | conventional | *Alternaria sp.* | MW776305 |  |  |  |  |  |  |
| E710 | winter wheat | Bamberka | root | conventional | *Periconia macrospinosa* |  |  |  |  |  |  |  |
| E711 | winter wheat | Bamberka | root | conventional | *Setophoma terrestris* | MW776306 |  |  |  |  |  |  |
| E712 | winter wheat | Bamberka | root | conventional | *Setophoma terrestris* | MW776307 |  |  |  |  |  |  |
| E713 | winter wheat | Bamberka | root | conventional | *Periconia macrospinosa* |  |  | OK328162 |  |  |  |  |
| E715 | winter wheat | Legenda | root | no till | *Epicoccum nigrum* | MW776308 |  |  |  |  |  |  |
| E716 | winter wheat | Legenda | root | no till | *Microdochium bolleyi* | MW776309 |  | OK328163 |  |  |  |  |
| E717 | winter wheat | Arkadia | root | no till | *Penicilium sp.* | MW776310 |  |  |  |  |  |  |
| E719 | winter wheat | Ostroga | root | no till | *Microdochium bolleyi* | MW776311 |  |  |  |  |  |  |
| E720 | winter wheat | Ostroga | root | no till | *Setophoma terrestris* | MW776312 |  |  |  |  |  |  |
| E721 | winter wheat | Bamberka | root | no till | *Fusarium oxysporum* | MW888908 |  |  |  | * |  |  |
| E723 | winter wheat | Bamberka | root | no till | *Setophoma terrestris* | MW776313 |  | OK328164 |  |  |  |  |
| E724 | winter wheat | Bamberka | stem | no till | *Periconia macrospinosa* | MW776314 |  | OK328165 |  |  |  |  |
| E726 | winter wheat | Euforia | root | no till | *Periconia macrospinosa* | MW776315 |  | OK328166 |  |  |  |  |
| E727 | winter wheat | Euforia | stem | no till | *Periconia sp.* | MW776316 |  |  |  |  |  |  |
| E728 | spring wheat | Rusałka | stem | no till | *Moesziomyces bullatus* | MW776317 |  |  |  |  |  |  |
| E729 | spring wheat | Rusałka | stem | no till | *Anthracocystis sp.* | MW776318 |  |  |  |  |  |  |
| E730 | spring wheat | Rusałka | seed | no till | *Anthracocystis sp.* | MW776319 |  |  |  |  |  |  |
| E731 | spring wheat | Rusałka | seed | no till | *Fusarium temperatum* | MW776320 |  |  |  | * |  |  |
| E733 | spring wheat | Rospuda | root | no till | *Setophoma terrestris* | MW776321 |  | OK328167 |  |  |  |  |
| E734 | spring wheat | Rospuda | stem | no till | *Alternaria sp.* | MW776322 |  |  |  |  |  |  |
| E735 | spring wheat | Rospuda | stem | no till | *Alternaria sp.* |  |  |  |  |  |  |  |
| E736 | spring wheat | Rospuda | stem | no till | *Alternaria sp.* | MW776323 |  |  |  |  |  |  |
| E737 | spring wheat | Kandela | root | no till | *Setophoma terrestris* |  |  | OK328168 |  |  |  |  |
| E738 | spring wheat | Bombona | root | no till | *Setosphaeria pedicellata* |  |  |  | * |  |  |  |
| E739 | spring wheat | Bombona | root | no till | *Penicillium olsonii* | MW776324 |  |  | * |  |  |  |
| E740 | spring wheat | Bombona | root | no till | *Fusarium oxysporum* | MW776325 |  |  |  | * |  |  |
| E741 | spring wheat | Bombona | root | no till | *Fusarium oxysporum* | MW776326 |  |  |  | * |  |  |
| E742 | spring wheat | Bombona | leaf | no till | *Meira sp.* | MW776327 |  |  |  |  |  |  |
| E743 | spring wheat | Bombona | leaf | no till | *Anthracocystis flocculosa* |  |  |  |  | * |  |  |
| E744 | spring wheat | Bombona | leaf | no till | *Penidiella sp.* | MW776328 |  | OK328169 |  |  |  |  |
| E745 | spring wheat | Bombona | leaf | no till | *Alternaria sp.* | MW776329 |  |  |  |  |  |  |
| E746 | spring wheat | Bombona | seed | no till | *Penicilium sp.* | MW776330 |  |  |  |  |  |  |
| E748 | spring wheat | Bombona | seed | no till | *Didymella sp.* | MW776331 |  |  | * |  |  |  |
| E749 | spring wheat | Bombona | seed | no till | *Gibellulopsis sp.* |  |  |  |  |  |  |  |
| E750 | spring wheat | Arabella | root | no till | *Fusarium sp.* | MW776332 |  |  |  |  |  |  |
| E751 | spring wheat | Arabella | root | no till | *Fusarium sp.* | MW776333 |  |  |  |  |  |  |
| E752 | spring wheat | Arabella | leaf | no till | *Penicilium sp.* |  |  |  | * |  |  |  |
| E753 | spring wheat | Arabella | stem | no till | *Fusarium sp.* |  |  |  |  |  |  |  |
| E754 | spring wheat | Arabella | stem | no till | *Fusarium solanii* | MW776334 |  |  |  | * |  |  |
| E755 | spring wheat | Arabella | seed | no till | *Cladosporium sp.* | MW776335 |  |  | * |  |  |  |
| E756 | spring wheat | Arabella | seed | no till | *Sarocladium sp.* | MW776336 |  |  |  |  |  |  |
| E757 | spring wheat | Arabella | seed | no till | *Talaromyces sp.* | MW776337 |  |  |  |  |  |  |
| E758 | spring wheat | Bombona | root | conventional | *Fusarium solanii* | MW776338 |  |  |  | * |  |  |
| E759 | spring wheat | Bombona | root | conventional | *Fusarium oxysporum* | MW776339 |  |  |  | * |  |  |
| E760 | spring wheat | Bombona | root | conventional | *Fusarium oxysporum* | MW776340 |  |  |  | * |  |  |
| E761 | spring wheat | Bombona | root | conventional | *Fusarium oxysporum* |  |  |  |  |  |  |  |
| E762 | spring wheat | Bombona | leaf | conventional | *Penicillium crustosum* |  |  |  | * |  |  |  |
| E763 | spring wheat | Bombona | stem | conventional | *Sarocladium sp.* |  |  |  |  |  |  |  |
| E764 | spring wheat | Bombona | stem | conventional | *Fusarium sp.* | MW776341 |  |  |  | * |  |  |
| E765 | spring wheat | Bombona | seed | conventional | *Sarocladium sp.* |  |  |  |  |  |  |  |
| E766 | spring wheat | Bombona | seed | conventional | *Alternaria alternata* |  |  |  |  | * |  |  |
| E767 | spring wheat | Rusałka | root | conventional | *Isaria farinosa* | MW776342 |  |  | * |  |  |  |
| E768 | spring wheat | Rusałka | root | conventional | *Fusarium oxysporum* | MW888909 |  |  |  | * |  |  |
| E769 | spring wheat | Rusałka | root | conventional | *Cladosporium sp.* | MW776343 |  |  | * |  |  |  |
| E773 | spring wheat | Rusałka | leaf | conventional | *Alternaria sp.* |  |  |  | * |  |  |  |
| E774 | spring wheat | Rusałka | leaf | conventional | *Aureobasidium pullulans* | MW776344 |  |  | * |  |  |  |
| E775 | spring wheat | Rusałka | leaf | conventional | *Alternaria sp.* | MW776345 |  |  |  |  |  |  |
| E777 | spring wheat | Rusałka | stem | conventional | *Stachybotrys bisbyi* |  |  |  |  |  |  |  |
| E778 | spring wheat | Rusałka | seed | conventional | *Stachybotrys bisbyi* | MW776346 |  |  |  |  |  |  |
| E779 | spring wheat | Rusałka | seed | conventional | *Penicilium sp.* |  |  |  |  |  |  |  |
| E780 | spring wheat | Rusałka | seed | conventional | *Penicilium sp.* | MW776347 |  |  |  |  |  |  |
| E781 | spring wheat | Rusałka | seed | conventional | *Penicilium sp.* |  |  |  | * |  |  |  |
| E782 | spring wheat | Rospuda | root | conventional | *Fusarium solani* |  |  |  |  | * |  |  |
| E783 | spring wheat | Rospuda | root | conventional | *Fusarium oxysporum* |  |  |  |  | * |  |  |
| E784 | spring wheat | Rospuda | root | conventional | *Penicilium sp.* |  |  |  |  |  |  |  |
| E785 | spring wheat | Rospuda | leaf | conventional | *Cladosporium allicinum* | MW776348 |  |  | * |  |  |  |
| E786 | spring wheat | Rospuda | leaf | conventional | *Alternaria sp.* | MW776349 |  |  |  |  |  |  |
| E787 | spring wheat | Rospuda | stem | conventional | *Alternaria infectoria* |  |  |  |  | * |  |  |
| E788 | spring wheat | Rospuda | seed | conventional | *Anthracocystis flocculosa* |  |  |  |  | * |  |  |
| E789 | spring wheat | Rospuda | seed | conventional | *Moesziomyces sp.* | MW776350 |  |  |  |  |  |  |
| E791 | spring wheat | Kandela | root | conventional | *Fusarium redolens* | MW888910 |  |  |  | * |  |  |
| E792 | spring wheat | Kandela | root | conventional | *Microdochium bolleyi* |  |  |  |  |  |  |  |
| E793 | spring wheat | Kandela | stem | conventional | *Alternaria sp.* | MW776351 |  |  |  |  |  |  |
| E794 | spring wheat | Kandela | stem | conventional | *Alternaria sp.* | MW888911 |  |  |  |  |  |  |
| E795 | spring wheat | Kandela | stem | conventional | *Alternaria sp.* | MW776352 |  |  |  |  |  |  |
| E796 | spring wheat | Kandela | seed | conventional | *Sarocladium sp.* | MW776353 |  |  |  |  |  |  |
| E797 | spring wheat | Arabella | root | conventional | *Setosphaeria pedicellata* |  |  |  | * |  |  |  |
| E798 | spring wheat | Arabella | root | conventional | *Fusarium oxysporum* |  |  |  |  | * |  |  |
| E800 | spring wheat | Arabella | root | conventional | *Fusarium oxysporum* |  |  |  |  | * |  |  |
| E801 | spring wheat | Arabella | leaf | conventional | *Fusarium oxysporum* |  |  |  |  | * |  |  |
| E802 | spring wheat | Arabella | stem | conventional | *Alternaria sp.* |  |  |  |  | * |  |  |
| E804 | spring wheat | Arabella | seed | conventional | *Fusarium oxysporum* | MW776354 |  |  |  |  |  |  |
